# Implementation of clinical practice guidelines using the Plan–Do–Study–Act framework: The methodology and experiences of the Academy of Nutrition and Dietetics Health Informatics Infrastructure Registry Study on gestational diabetes mellitus

**DOI:** 10.1002/ncp.70043

**Published:** 2025-10-02

**Authors:** Kerri Lynn Knippen, Lindsay Woodcock, Constantina Papoutsakis, Diana M. Gonzales‐Pacheco

**Affiliations:** ^1^ Department of Public and Allied Health Food and Nutrition Program, Bowling Green State University Bowling Green Ohio USA; ^2^ Data Science Center, Research, International, and Scientific Affairs (RISA), Academy of Nutrition and Dietetics Bowling Green Ohio USA; ^3^ Nutrition and Dietetics, College of Education and Human Sciences, University of New Mexico Albuquerque New Mexico USA

**Keywords:** Academy of Nutrition and Dietetics Health Informatics Infrastructure, gestational diabetes mellitus, implementation science, medical nutrition therapy, Nutrition Care Process, quality improvement, registered dietitian nutritionist

## Abstract

**Background:**

Registered dietitian nutritionists (RDNs) use clinical practice guidelines (CPGs) to inform evidence‐based practice. Despite the availability of CPGs, guidelines are not always translated into practice. This challenge is central to implementation science (IS), which seeks to understand how evidence can be adopted and sustained.

**Methods:**

The Gestational Diabetes Mellitus (GDM) Registry Study was a multiphase, multisite hybrid implementation study that explored guideline implementation using quality improvement (QI) methods grounded in the Model of Improvement and guided by Plan–Do–Study–Act (PDSA) cycles. Following a baseline period, RDNs completed training, conducted a gap analysis, and identified 2 CPG implementation aims. Sites completed iterative PDSA cycles. Deidentified nutrition care data were entered into the GDM Study Registry and manually audited to evaluate process outcomes. RDNs participated in a closing interview. Qualitative data were analyzed using a constructivist approach and reflexive thematic analysis, supported by artificial intelligence (AI)–assisted qualitative software.

**Results:**

Six themes were identified, highlighting the value of assessing current practices and the flexibility of PDSA as an implementation strategy. Themes were mapped against the Normalization Process Theory and Consolidated Framework for Implementation Research and showed alignment between PDSA and implementation principles. The registry audit demonstrated improved process measures. The median normalization score across RDNs (9.00) and sites (9.42) was high, suggesting normalization.

**Conclusion:**

PDSA facilitated the work of normalization and enabled practice changes. This study contributes to IS by demonstrating how QI strategies, such as PDSA can help RDNs translate evidence into everyday nutrition care.

## INTRODUCTION

Evidence‐based practice (EBP) is fundamental to optimizing patient‐centered, science‐based nutrition care, enhancing credibility, fostering standardization, and improving quality and effectiveness.^1^ Clinical practice guidelines (CPGs) are systematically developed to translate research into standardized recommendations, providing an EBP foundation for practitioners.^2^ Evidence‐based nutrition practice guidelines (EBNPGs) are unique CPGs, as they incorporate the Nutrition Care Process (NCP) to improve patient outcomes.^3^, ^4^ Although CPGs can serve as a bridge between research and real‐world application, dissemination alone does not lead to successful implementation or improved patient outcomes.^5^

The “know‐do” gap, in which research fails to translate into practice, remains a significant challenge in nutrition care.^6^ Translating CPGs from knowledge to action is complex, multifactorial^7^ and influenced by barriers, including environmental context, lack of systems—processes and resources, resistance to change, inadequate training, skills, knowledge, and guideline utility.^8^, ^9^, ^10^

Research underscores that improving CPG uptake depends on context, specifically understanding where and how evidence is put into practice.^11^ There is a need to expand implementation science (IS) in nutrition,^12^, ^13^ as IS aims to improve the uptake of EBP into routine care by closing the gap^14^ between what is known and what is practiced.^15^ Successful guideline implementation requires multilevel strategies^16^ tailored to local needs, including the use of performance gap assessments, education, clinical decision support, point‐of‐care alerts, and feedback‐audit mechanisms.^8^ Leadership, organizational support, capacity building, and stakeholder engagement are needed to create a culture of quality that celebrates EBP and adherence to CPGs.^6^, ^8^, ^9^

Given the importance of context in IS, there have been calls to integrate quality improvement (QI) and IS efforts within healthcare.^17^, ^18^, ^19^ Although QI and IS are distinct, they have similar goals.^17^ QI methodology offers a structured and systematic framework to implement practice changes at the local level.^20^, ^21^ Common QI models include the Model for Improvement, Lean, and Six Sigma. The Model for Improvement is a structured approach to continuous QI that is guided by defining aims, outcomes, and change strategies, which are tested using Plan–Do–Study–Act cycles (PDSA).^22^ PDSA offers an iterative approach to testing changes and enabling small‐scale improvements over time.^22^, ^23^ PDSA can be used with other QI models, such as Lean and Six Sigma, which respectively focus on reducing waste and minimizing variation within processes.^24^

This report presents the methodology and insights from the “GDM Registry Study,”^25^ which was grounded by the Model for Improvement and used the PDSA method as an implementation strategy to drive small‐scale changes in the uptake of CPGs for gestational diabetes mellitus (GDM) among registered dietitian nutritionists (RDNs). Although PDSA is widely used to improve quality, there are gaps in its application,^24^ including the use of PDSA for implementing CPGs. A strength of PDSA is the ability to address context in each step of the cycle.^26^ Therefore, using QI methods, such as PDSA, within implementation efforts has the potential to promote contextually relevant and sustainable changes to support guideline uptake and adherence. The aim of this study was to report the use of PDSA cycles as a pragmatic approach to support RDN‐led implementation of GDM guidelines in a variety of clinical settings.

## METHODS

### Study design

The hybrid implementation study was approved by the Institutional Review Board (IRB) of Bowling Green State University (Approval No. 1626498). External clinical sites in the United States were recruited and sites had the opportunity to cede IRB review to Bowling Green State University's IRB or obtain local IRB approval. The reporting of qualitative data in this report was guided by the Standards for Reporting Qualitative Research (SRQR) to ensure transparency.^27^

**Figure 1:**
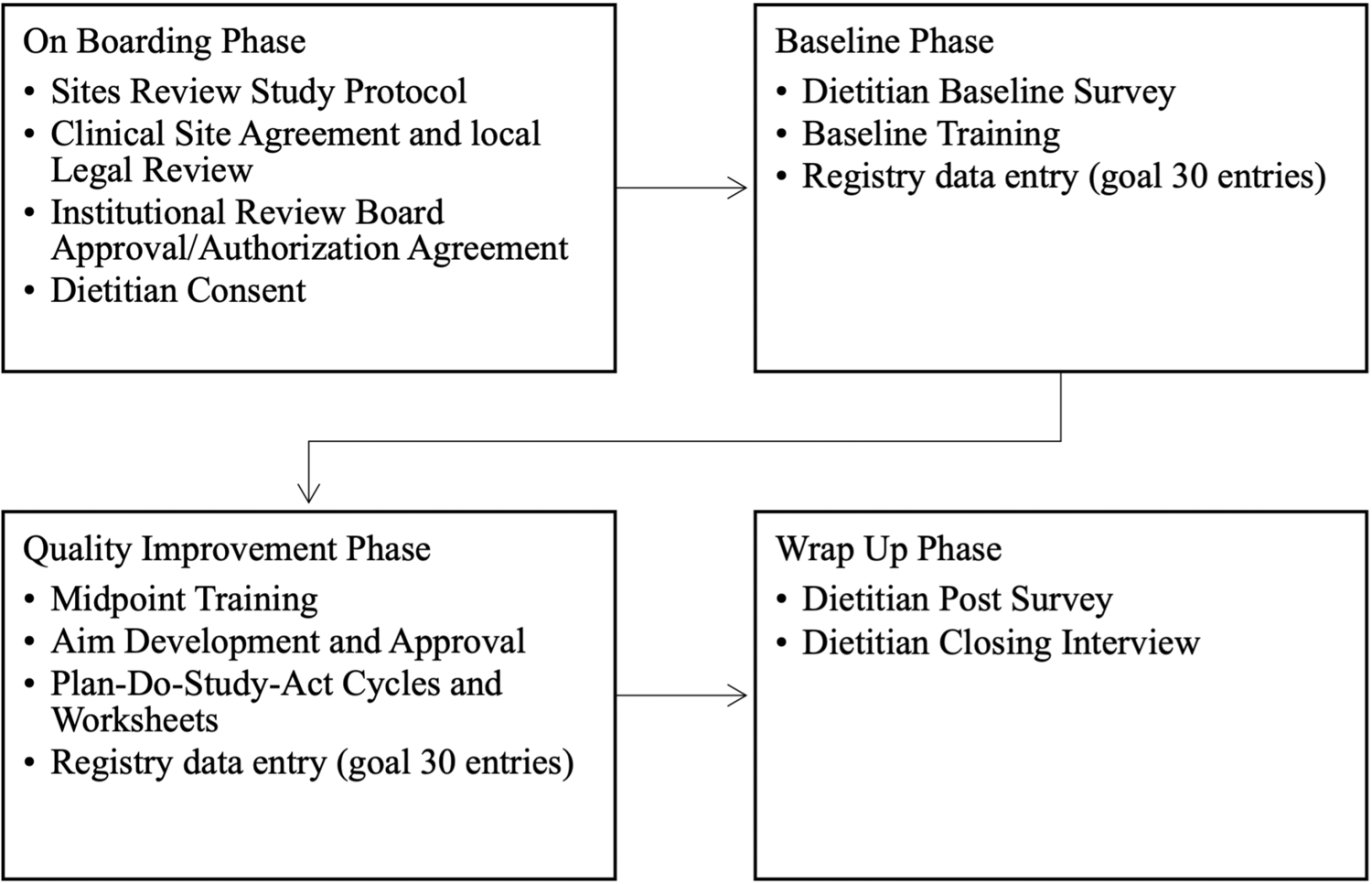
Overview of study phases and activities for dietitians in the GDM Registry Study.

The GDM Registry Study included four phases, with the baseline period serving as the control (Figure 1). The project spanned 3 years, starting with methodology development in July 2020 and data collection ending in February 2023. The guideline implementation intervention was delivered to external clinical sites where RDNs provided medical nutrition therapy (MNT) for GDM patients. The implementation strategy consisted of RDN training and a structured QI phase, in which RDNs used the PDSA framework to apply change strategies for the uptake of CPGs for GDM. A deidentified data registry was used by RDNs to document nutrition care indicators for eligible patient encounters over the course of the study.

### Participants

The study included two participant groups: (1) RDNs at participating clinical sites and (2) patients with GDM. All participants consented only after procedures were explained and questions were answered. Dietitians completed an electronic written consent. The IRB approved a waiver of written consent for patients; a verbal consent process was used as written consent would have been the only information available to the researchers linking a patient to the study.

Dietitians had to be working in a clinical setting providing MNT for GDM with sites having a minimum volume of 10 GDM encounters per month. Verification of RDN registration status with the Commission on Dietetic Registration (CDR) was required. Licensure was confirmed for RDNs practicing in states with licensure requirements. Credentials were verified using free, online, and publicly accessible search queries from CDR and state licensure boards. Multiple RDNs could participate from the same site. Participation also required a clinical site agreement to be on file. Dietitians were excluded if credentialing information was not provided, if credentials could not be verified, or if a clinical site agreement was not in place.

Patient eligibility consisted of current diagnosis of GDM defined by *International Classification of Diseases* (*ICD‐10*) code on the referral for initial MNT assessment, age >18 years, and residence in the United States. Patients who had already received MNT for GDM during the current pregnancy but were not enrolled at their initial assessment were not eligible. Patient participation continued through 12 weeks postpartum.

### Recruitment

#### Dietitians‐sites

**Figure 2:**
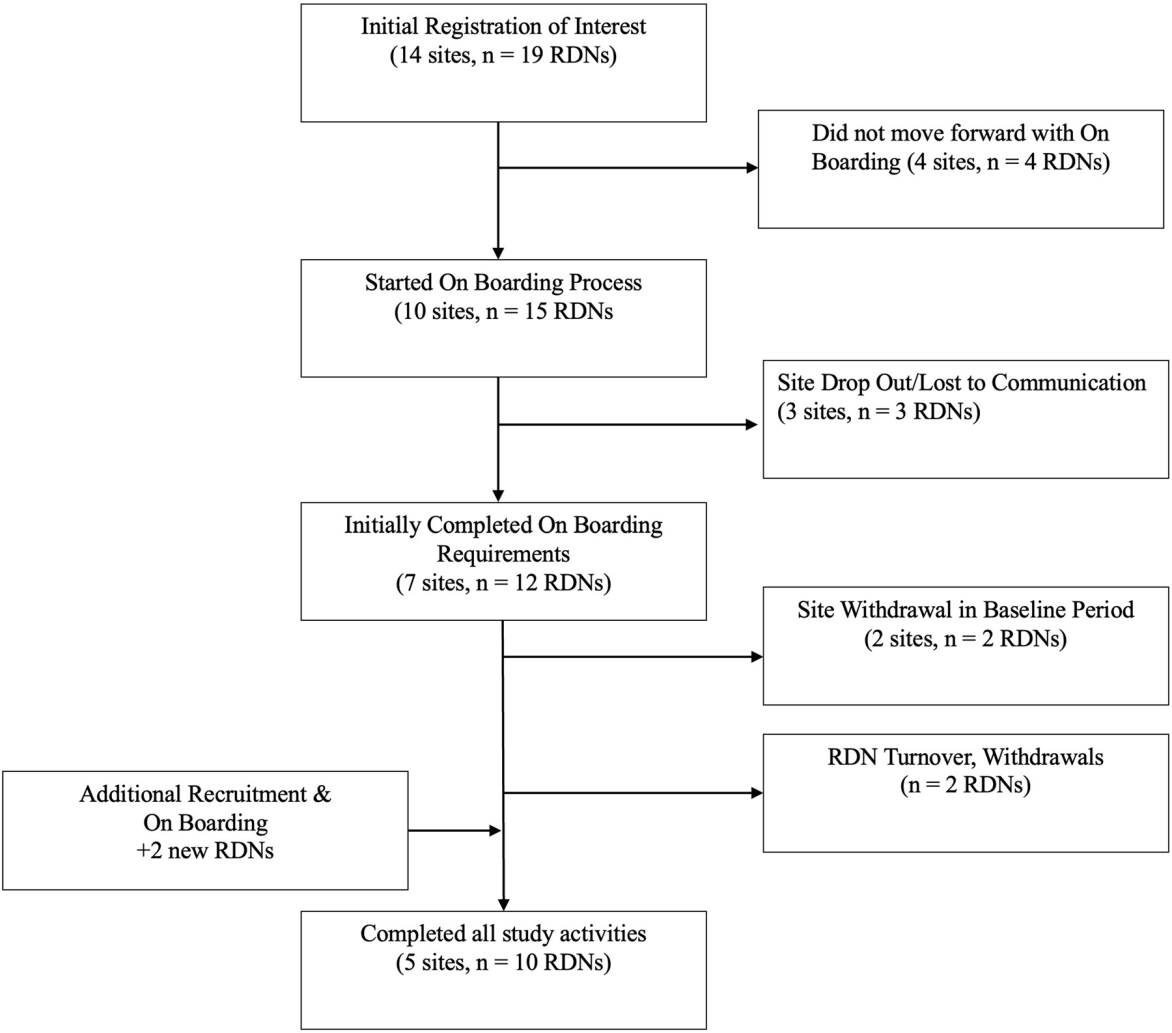
Flow diagram describing registration, onboarding, and sample of dietitians who completed the GDM Registry Study. RDNs, registered dietitian nutritionists.

Sites and RDNs were recruited through various Academy of Nutrition and Dietetics (Academy) outreach channels, including email, professional listservs, and social media posts. Virtual information sessions, each lasting 30 min, were conducted and RDNs and/or sites were invited to register. A total of 14 sites initially registered, seven sites completed the onboarding process, and five sites completed all study activities (Figure 2). After site onboarding, RDNs were invited and completed the electronic consent. Two sites withdrew: one after enrolling a single patient, citing personal reasons, and the other prior to recruitment because of decreased patient volume, which made study targets unachievable. As a result of employee turnover (unrelated to the study), two RDNs were withdrawn and two new RDNs were onboarded.

Dietitians could earn up to 9.5 h of continuing professional education (CPE) units for completing study training. Clinical sites were eligible to receive a financial incentive for meeting study milestones, either as electronic Amazon gift cards (up to five–$100 gift cards) distributed throughout the study or as a one‐time payment of up to $500 at the study's conclusion. Financial incentive was delivered to the site's financial designee, and it was the site's discretion to determine how the incentive would be used locally.

#### Patients

A convenience sampling approach was used and RDNs invited eligible patients during their initial MNT encounter for GDM. Patients received a study letter, information sheet, registry privacy details, and were given time to review materials and ask questions. Verbal consent was obtained using a standardized process and documented in the medical record. Patient participation consisted of receiving routine nutrition care, as determined by each clinical site. Consent allowed RDNs to enter nutrition care data into a study registry.

### Implementation strategies and resources

The study used several implementation strategies to facilitate the uptake of CPGs, including the training of RDNs. Dietitians completed seven asynchronous training modules that were hosted on the Academy's Learning Management System. Five modules were previously developed by the Academy; the study orientation and QI modules were created by the study's principal investigator (PI) (K.L.K.) and reviewed by two independent experts. All modules were approved for CPE from CDR.

Baseline training included four modules that covered study procedures, ethics, NCP, and registry use. Dietitians received a study manual, job aide, and sample case studies. Each site also participated in a virtual session with the PI for questions and a live registry tutorial.

Dietitians had access to a secure, online portal that included study documents, materials, announcements, and a discussion forum. They received a complimentary subscription to the Academy's electronic Nutrition Care Process Terminology (NCPT) platform used by the registry^28^ and could request additional meetings with the study PI.

Midpoint training included three modules on GDM nutrition practice guidelines and QI, focusing on aim development, outcome measures, and PDSA cycles. PDSA was selected because of its straightforward structure and flexibilty.^26^, ^29^ Unlike other QI methods, it requires minimal training, resources, and leadership support. PDSA's quick, adaptable cycles made it well‐suited for the multisite GDM Registry Study, in which RDNs operated in diverse settings with varying QI experience. Dietitians received a complimentary subscription to the Academy's Evidence Analysis Library (EAL) for guideline summaries, recommendations, evidence grading, and conditions of applying the recommendations.^30^

After the midpoint training, sites were required to meet with the PI. Dietitians received a guideline implementation and QI manual developed by the study PI and reviewed by the study's advisory experts (see Supplemental File 1). The manual supported the transition to the QI phase and covered the Model for Improvement, developing QI aims, PDSA cycles, identifying outcomes and creating a data collection strategy, key drivers, worksheets based on PDSA and Normalization Process Theory (NPT),^31^ as well as a gap analysis assessment and guideline implementation checklist.

Sites used the gap analysis to explore current GDM practices, roles, communication processes, and resources needed to implement a CPG recommendation. Sites could generate or request a baseline registry report to support the analysis.

The gap analysis informed aim development. Each site submitted two aims using an aim worksheet that included elements recommended by the Institute for Healthcare Improvement, outlining the problem, goal for improvement, timeline, and improvement strategies.^32^ The PI reviewed the aims for clarity, alignment with CPGs, feasibility, and measurability. A listing of aims, their relationship to guidelines,^33^, ^34^, ^35^, ^36^ and examples of process outcomes is shared in Table 1.

**Table 1:** Aims, guideline alignment, and PDSA outcomes for quality improvement phase for sites who completed all study activities of the GDM Registry Study.

Site	PDSA aims	Guideline alignment	Examples of process outcomes
Site A	Counsel 100% of patients on protein needs and sources of protein at initial visit	Macronutrient requirements^a^	% patients with protein education and counseling documented in intervention
	Counsel 100% of patients to self‐monitor protein sources and intake so data can be used for assessment/reassessment	Macronutrient requirements^a^	% patients with assessment/reassessment of estimated protein intake
Site B	75% of patients will see RDN within 3 weeks of referral	Referral for MNT^a^	% patients with MNT in <3 weeks of referral (site selected timeline) (document time from referral to MNT in # weeks under the “patient, client chief nutrition complaint”)
	75% of patients will be scheduled to see diabetes educator within 12 weeks of delivery	Prevention of recurrence of GDM or type 2 diabetes^b^	% patients with postpartum diabetes education visit scheduled (site to document scheduling of the postpartum visit under intervention)
Site C	100% of patients will be asked about intention to breastfeed at initial visit	Breastfeeding promotion,^b^, ^c^	% patients with breastfeeding intention assessed
	100% of patients will receive counseling on the barriers–benefits of breastfeeding in relation to T2DM prevention	Breastfeeding promotion,^b^, ^c^	% patients with breastfeeding guidance (benefits–barriers) documented in the intervention
Site D	60% of patients will submit accurate self‐monitoring food and blood glucose logs to improve individualization of carbohydrate prescription	Carbohydrate prescription^a^; Nutrition Assessment—Food and nutrition‐related history^a^	% with food–blood glucose logs completed at reassessment with carbohydrate data recorded
	90% of patients will be instructed on weight gain recommendations based on the Institute of Medicine (IOM) guidelines for pregnancy	IOM weight gain guidelines^d^; Nutrition Assessment—Anthropometrics^a^	% with relevant anthropometrics documented in assessment; % patients where weight change was reassessed at subsequent encounters; % receiving counseling on strategies that may promote appropriate weight gain
Site E	Increase percent of patients receiving assessment of protein intake as well as guidance on protein recommendations, protein prescription	Macronutrient requirements^a^	% patients with protein assessment; % patients where protein education documented in intervention; % patients with protein prescription; % with estimated protein intake at reassessment
	Increase percent of patients receiving physical activity assessment and education on physical activity guidelines appropriate for pregnancy and GDM	Physical activity guidance^a^	% patients where RDN completed assessment of physical activity (physical activity history, type, duration, frequency); % patients where intervention documents physical activity guidance (ie, type, duration, frequency, safety precautions)

Abbreviations: GDM, gestational diabetes mellitus; IOM, Institute of Medicine; MNT, medical nutrition therapy; PDSA, Plan–Do–Study–Act; RDN, registered dietitian nutritionist; T2DM, type 2 diabetes mellitus.

^a^ Academy of Nutrition and Dietetics. Evidence‐Based Nutrition Practice Guidelines for Gestational Diabetes, 2018.^33^

^b^ Academy of Nutrition and Dietetics. Evidence‐Based Nutrition Practice Guidelines for Gestational Diabetes, 2008.^34^

^c^ American Diabetes Association. Standards of Clinical Care. Pregnancy, 2022.^35^

^d^ Institute of Medicine. Weight Gain Guidelines for Pregnancy, 2009.^36^

Sites identified change drivers and prepared for at least two PDSA cycles. For each cycle, they submitted a worksheet outlining their aim, predictions, planned changes, and data collection strategy.^37^ The PI reviewed submissions for feasibility, registry compatibility, and alignment with study goals, providing guidance as needed. After each cycle, sites submitted a summary of outcomes and next steps (adopt, adapt, abandon). QI data came from observations, site records, and registry extracts. The PI reviewed summaries and offered suggestions for future test cycles.

### Data collection

The study included quantitative and qualitative data collection to broadly explore CPG uptake. Dietitians completed a presurvey‐postsurvey to evaluate changes in knowledge, confidence, skill, and attitudes toward EBP, QI, and implementation. A data registry was used by RDNs to document deidentified nutrition care data for patients who consented; details are in the “Data Registry” section.

Sites submitted aim and PDSA worksheets, which provided context for understanding barriers, solutions, and PDSA outcomes. Dietitians completed a concluding interview with the PI. Interviews were guided by a semistructured interview guide developed by the PI to explore what changes were made, what supported implementation, key players, communication, perceived normalization, barriers, and future intentions. Additional probes were used to elicit clarification and elaboration. Interviews were conducted via telephone or via a virtual call in option. Audio data were collected and deidentified transcripts were generated.

### Data registry

Dietitians used the “GDM Study Registry” to document indicators of nutrition care using standardized NCPT.^38^ The registry was maintained through the Academy of Nutrition and Dietetics Health Informatics Infrastructure (ANDHII), an online, secure tool for data collection and management.^1^, ^39^ ANDHII allows RDNs to document deidentified NCP data using the NCPT to describe the care provided to the patient. Data entry is organized by the NCP Model.^40^ ANDHII includes a “smart visit” component that allows RDNs to quickly identify and select NCPT relevant to the encounter. The use of standardized language across the NCP facilitates aggregate process and outcome evaluation.^41^, ^42^

Two RDNs with expertise in GDM and nutrition practice guidelines for GDM were consulted during the registry setup. Although the NCPT includes >2300 unique terms, custom terms were developed for the registry project. Custom terms were based on expert opinion of the advisory experts, the study PI, ANDHII staff, and the GDM nutrition practice guidelines.

Dietitians sequentially entered deidentified nutrition care encounters into the registry, and no protected health information was recorded. A random reidentification code generated by the registry was secured in the patient's medical record. The research team never had access to any identifiers to link the patient to the reidentification code. Dietitians were asked to enter data in real‐time alongside completion of their medical record entry. Subsequent nutrition care encounters for GDM were entered through 12 weeks postpartum.

Early in data collection, the study PI observed gaps in the consistency of assessment data entry across sites. To improve data quality, a standard assessment template was created for new patient entries, prompting RDNs to enter limited demographic data and relevant patient‐family‐client history. All fields were optional except for the RDN and patient reidentification codes, and RDNs could add additional assessment data as needed.

### Data analysis

#### Qualitative data

We used a constructivist approach and reflexive thematic analysis (RTA) method to analyze the qualitative interview data.^43^ This approach was selected as it recognizes that experiences are dependent on context and the goal was to understand how RDNs experienced the PDSA cycles and how strategies, such as PDSA, influenced implementation. RTA has been used previously to understand views and experiences of healthcare providers and to describe barriers and facilitators of implementation.^44^, ^45^, ^46^

RTA also recognizes the value of the researcher's lens or positionality in interpreting the data.^43^ In this case, all interviews were conducted by the PI who has extensive experience with GDM care as an RDN, diabetes educator, and researcher. The PI played an active role in the research, from providing technical assistance and training to reviewing aims and PDSA worksheets. The PI was familiar with each site's workflow, successes, and challenges they experienced during the project. Collectively, these perspectives were valuable to construct meaning from the data.

The study PI read all interview transcripts to gain familiarity with the text. The PI reviewed all aim and PDSA worksheets to supplement understanding of each site's QI phase. Reflexive notes were recorded and the coding process began. The PI completed three rounds of manual coding using Microsoft Word and Excel. Initial codes were primarily semantic and inductively drawn from transcripts. Latent codes were later developed. Codes were subsequently defined and organized in a codebook. Codes were clustered based on their relevance to the QI cycle (ie, understanding contextual factors, exploring and preparing, planning, doing, studying, and acting). Final themes were developed by the PI to represent insights and experiences across sites and RDNs in the study. Theme labels and descriptions were iteratively refined to enhance clarity with the assistance of ChatGPT (OpenAI, GPT‐4)^47^ and final wording was determined by the PI. The final themes were organized in the researcher‐derived codebook (see Supplemental File 2).

To support analytic triangulation, AILYZE, an artificial intelligence (AI)–powered qualitative analysis tool^48^ was used to generate two independent thematic analyses: one based on AI‐generated coding and another using the researcher‐developed codebook. We compared the two AI thematic analyses with our coding. This process augmented our analysis to explore alignment of coding and enhance analytic reflexivity. To strengthen our interpretation, the final themes were mapped onto constructs from the NPT^31^ and the Consolidated Framework for Implementation Research (CFIR).^49^, ^50^

#### Registry data

The PI and ANDHII staff reviewed registry data monthly for quality control and site feedback. Errors (eg, duplicates, incomplete entries) were removed after site consultation. The PI also sent monthly summaries to sites and audited data to monitor QI progress.

## RESULTS

A total of 10 RDNs from five clinical sites completed all study activities (Table 2). The majority identified as female, non‐Hispanic, and White. Dietitians were working in a variety of clinical settings and had variable experience. At baseline, RDNs had been in practice seven months to 24 years and had worked in their current setting from 1 month to 11 years; of which several (*n* = 4) had been in their current role for 3 months or less. Experience in GDM care varied, ranging from 2 months to 10 years, with two RDNs reporting 6 months or less of GDM specific experience. Most held a graduate degree and an advanced specialty credential in diabetes. The duration of holding the diabetes credential ranged from 1.5 to 15 years.

**Table 2:** Characteristics of participating dietitians and study sites for the GDM Registry Study.

	*n* (%)
*Number GDM patients RDN sees per month*	
<5	1 (10%)
5–10	3 (30%)
11–14	3 (30%)
15–19	1 (10%)
20–29	1 (10%)
30+	1 (10%)
*Setting*	
Hospital outpatient clinic	4 (40%)
Maternal fetal medicine outpatient clinic	4 (40%)
Diabetes self‐management clinic	2 (20%)
*Education*	
4‐year degree	1 (10%)
Master's degree	9 (90%)
*Additional credentials*	
CDCES	6 (60%)
BC‐ADM	1 (10%)
Other	2 (20%)
*Sex reported*	
Male	1 (10%)
Female	9 (90%)
*Race*	
White	7 (70%)
Asian	1 (10%)
Other	2 (20%)
*Ethnicity*	
Hispanic, Latino	1 (10%)
Non‐Hispanic	9 (0%)

*Note*: Data based on dietitians and sites who completed all study activities.

Abbreviations: BC‐ADM, Board Certified Advanced Diabetes Management; CDCES, Certified Diabetes Care and Education Specialist; GDM, gestational diabetes mellitus; RDN, registered dietitian nutritionist.

At baseline, most RDNs reported limited familiarity with the Academy's EAL, with 10% indicating they were not knowledgeable and 60% reporting they were only slightly knowledgeable. Similarly, use of the Academy's EBNPG for GDM was infrequent: 60% reported never using the guidelines, 10% rarely, 10% sometimes, and only 20% reported using them often in GDM care.

### Qualitative results

The qualitative analysis explored RDN experiences using PDSA cycles to implement CPGs in practice. Initially, 52 codes were generated and clustered into six themes. During the second and third rounds of manual coding, codes were organized and further consolidated for a total of 28 codes under six themes. The AI‐generated thematic analysis described three themes (effective communication and collaboration, implementation, and logistical challenges) and 12 subthemes, which highlighted team dynamics, roles, shared goals, structured processes, time, training, resources, adaptability, and integration. When our codebook was applied, the AI tool demonstrated continuity with manual coding and augmented our analysis and analytic reflexivity. The six themes identified from the RTA are thematically presented in this report with quotes to illustrate RDN experiences. For more information, please view the codebook (see Supplemental File 2).

#### Navigating context, capacity, and possibility

Dietitians described how their roles, systems, and constraints impact their daily work and how these factors shape implementation. Navigating context required RDNs to balance their aims within the constraints they faced. Autonomy allowed sites to adapt the work to their setting and prioritize changes that mattered to them. In some ways, autonomy limited opportunities for growth and improvement. Several participants described how they chose something “tangible” that they could do “without relying on other people.” The extent of stakeholder engagement varied by site and PDSA aim. Some sites engaged with other stakeholders, such as but not limited to nursing, support staff, physicians, and other specialists. Often stakeholders were supportive but from a distance. Factors outside the system, such as insurance, were also barriers to aim selection.

There was a common thread that time and communication were barriers to changing clinical practice. The current workflow was heavily centered around patient care, leaving little to no time for discussions that could facilitate improvements. Although communication was sometimes suboptimal and reliant on email, RDNs adapted and several noted that the project's structured efforts ultimately enhanced communication. These experiences demonstrate that although communication may be difficult, structured efforts can create opportunities to improve patient care without disrupting workflow.

Despite limitations, RDNs recognized that if practices remained unchanged, this would negatively impact patient care. Participants expressed a proactive attitude and found ways to adapt and challenge the status quo. For example, one RDN stated, “I know that it's important not to just rest on your laurels or go with a status quo of what you always have been doing.” This reflects an awareness of the pitfalls of complacency in clinical practice.

#### Sizing up change: Fresh eyes on familiar practices

Participants emphasized the importance of clear communication, collaboration, and evidence to support guideline implementation. Strength of data supporting guidelines compared with anecdotal practice experiences sometimes challenged confidence to implement guidelines. One RDN expressed such concern stating, “I wish there was a little bit stronger data.” The presence of multiple guidelines from organizations also created confusion at times. Dietitians expressed the need for structured communication and regular updates to stay on top of guidelines. Dietitians described how the study helped them reevaluate their practices and the importance of revisiting foundational knowledge, such as the NCP.

Dietitians described that although there is a focus on improvement in their organizations, there was a lack of regularity in assessing practices. The synthesis of qualitative data highlighted the importance of structured assessment as a critical step toward improving EBP. As one participant mentioned, “It's been great to have this as a tool [referring to assessment] to help show what we're missing and where we want to improve and just talk about where the gaps might be.” It was important for sites to create space for honest reflection to consider context, fit, roles, resources, and timeline. For example, one RDN stated, “It's kind of like you look at all these different interventions and have these big ideas, but then you also kind of have a humbling conversation of, what can we actually do? What's realistic? What do we have control over?” The individual and collective commitment to self‐assessment was evident and underscores the significance of revisiting familiar practices with fresh eyes to identify gaps and opportunities.

#### Plan phase: Translating shared vision to action

Overall, the synthesis of data regarding the planning phase revealed the significance of using structured planning tools as a critical step toward implementation. The planning process helped sites find a shared understanding as they transitioned into the QI phase. Most sites naturally identified a champion within their team who could “spearhead” the QI phase. This was particularly important when sites had multiple RDNs and/or RDNs were working in different locations.

Clear frameworks and resources (eg, QI manual, PDSA) to guide efforts were valued by participants. Participants emphasized the importance of aim‐development activities and PDSA worksheets. For example, one stated, “I do think it's helpful knowing where we're going and what our goal is.” Setting clear, concrete aims helped sites translate their vision into preparing for implementation. Dietitians discussed how they used the NCPT to think about outcomes that could be assessed in the registry; an approach that was highlighted in the training and QI manual, making its application in practice noteworthy.

PDSA helped RDNs operationalize change or “get down to details and the actual what's next steps.” Tools enhanced communication and collaboration among team members, ensuring that everyone was aligned in their efforts to achieve a shared vision. For example, one participant shared their experience, “talking to the team about where we think our strengths are already and then what we want to work on. Everyone agreed that yes, we want our GDMs to see the RDN as a priority and we want to get them back in for follow‐up, so we all agreed on that together.”

#### Do phase: Boots on the ground and eyes to the sky

During the “Do” step, sites were engaged with boots on the ground and eyes toward the sky—meaning as they did the work, they kept sight of their goals. Dietitians navigated “on the ground” challenges while staying patient‐centered. PDSA helped facilitate the “do” phase by breaking complex challenges into manageable changes. A summary of PDSA worksheet findings, change strategies, barriers, and observations is provided in Table 3.

**Table 3:** Change strategies, barriers, and observations extracted from PDSA worksheets completed by dietitians participating in the GDM Registry Study.

Focus of aim	Change strategies	Barriers and RDN observations
MNT referral	Coordinated with administrative staff to prioritize GDM referrals (eg, initiated Epic urgent holds, waitlist created) Improved communication processes New workflow: staff contacted patient within 3 days Offered virtual visits as needed	Limited RDN availability Administrative staff turnover Scheduling inefficiencies
Postpartum visit to prevent recurrence of **GDM or** T2DM development	New workflow: integrate postpartum visit as part of standard care for GDM referral; schedule postpartum visit at initial contact; reminders at 36‐week visit and group classes Administrative staff trained to schedule visit and document information in Epic	Staff changes disrupted scheduling Required regular communication among team which was discussed at staff meetings Limited time to address importance of postpartum visit at initial visit Patient reluctance to schedule postpartum visit
Macronutrient Requirements (Protein)	Integration of new nutrition education tools: handout from the *Nutrition Care Manual*; protein portion size visuals Integration of assessment/reassessment of protein food servings estimated in 24 h or protein estimated intake per kilogram body weight in 24 h RDNs encouraged protein self‐monitoring on food–blood glucose logs RDNs integrated protein in nutrition prescription	Many patients below recommended protein intake at baseline Limited awareness of protein needs among patients at initial MNT visit Recall limitations (eg, protein amount); collection of monitoring data improved with log use
Individualize carbohydrate prescription	Deimplemented prior glucose monitoring log; communication with staff and RDN removed from all sites Developed new log; staff input Trained staff on new food intake–blood glucose monitoring log RDNs encouraged self‐monitoring at visits Communication with RDNs and other staff to ensure logs distributed at initial visit and collected at subsequent visit(s)	Logs not consistently returned and inaccurate logs hindered assessment–reassessment Some patients preferred digital tools Logs led to tailored education at follow‐up visits (eg, portion size recommendations for specific foods, label reading) Logs facilitated medication initiation and titration
Breastfeeding promotion	RDN integrated assessment of breastfeeding intention at initial visit; assessed breastfeeding support and barriers for breastfeeding RDN identified and encouraged lactation consultant as resource Integrated breastfeeding assessment and guidance prompts into the EMR nutrition note template	Patients generally receptive to breastfeeding discussion
Physical activity guidance	RDN assessed physical activity at each visit Integrated educational handout on physical activity	Difficulty estimating physical activity Challenges quantifying goal achievement
Gestational weight gain	RDN educated other clinicians on IOM guidelines Integration: created smart phrases for EMR and integrated handouts on gestational weight gain into EMR Communication with staff during PDSA cycles	Late referrals, patients already at or exceeded gestational weight gain recommendations Focus shifted to slow rate of weight gain (eg, physical activity guidance, food–nutrient intake) Nurse practitioners valued RDN role in weight counseling

Abbreviations: EMR, electronic medical record; GDM, gestational diabetes mellitus; IOM, Institute of Medicine; MNT, medical nutrition therapy; PDSA, Plan–Do–Study–Act; RDN, registered dietitian nutritionist; T2DM, type 2 diabetes mellitus.

A defining feature of PDSA is its focus on small‐scale changes. Dietitians described meaningful shifts in practice and how the iterative process allowed them to build on early results. Several described how they began to evaluate their nutrition care against guidelines during the PDSA cycle. They adapted their approaches while staying grounded in patient‐centered care. For instance, one site initially focused on adhering to pregnancy weight gain guidelines, but after recognizing that many patients had already exceeded targets by their first MNT visit, they shifted their aim to slowing the rate of gestational weight gain through MNT.

There was a shared recognition that achieving EBP is an ongoing progress, reinforcing the iterative nature of change, as one RDN noted, “we are still working on those barriers,” and another expressed that there is more work to do, saying “we have not been able to implement all the changes that we have come to learn or want to address yet.” A third remarked, “I think the study has really helped us realize that this is a focus that we can just keep working on until we see that it's working.” These comments reinforce how RDNs recognized the continuous nature of improvement and cumulative impact over time.

Despite successes, RDNs described communication challenges, with many expressing a need for more in‐person communication. Data revealed practical strategies for doing the work of guideline implementation, including access to clear, comprehensive guidelines, team support, communication, and using structured approaches like PDSA.

#### Study phase: Learning from data and experiences

The “Study” step allowed sites to reflect and assess whether changes were leading to outcomes. Early results, internal team feedback, audits of their own charts, and registry audit data were used by sites to assess achievement of aims. Several RDNs commented on the benefits of using NCPT and the registry to document outcomes and how structured language helped with team cohesion.

The study step provided a check‐in and fostered accountability. For example, one RDN noted, “We're kind of scheduling an appointment with ourselves…study provided a lot of structure, which in just daily practice it's easy to let go of that if you don't have those prompts.” Feedback was incorporated into planning the next PDSA cycle, as one shared how they used PDSA to adjust for future improvement, “Yeah, I think it's [referring to PDSA] good because you can kind of adjust and build on what you're already doing…seeing what works or how to make it better I think was helpful.”

#### Act phase and beyond: Seeing the ripple, strengthening the system

Lastly, the “Act” step prompted sites to reflect on next steps using what they learned to refine or expand changes. Several sites discussed how they embedded change, including integrating education resources, developing guideline‐aligned reminders or templates, or including phrases for electronic medical record documentation. Embedded changes reinforced guidelines, strengthened the system, and supported broader dissemination. As one RDN explained, “I think it definitely helped to add it into our note template… that's kind of the outline I have in my head when I'm going through with my consults.” Another described how changes to the template spread the message to their nurse diabetes educator colleagues.

Dietitians expressed a forward‐thinking mindset, with many thinking about integration and using PDSA beyond the scope of this project. One RDN described a future strategy to integrate a checklist related to standards of care, whereas others expressed interest in sharing what they have learned through system‐wide dissemination efforts. Encouragingly, many discussed their intention to continue QI efforts using PDSA in the future. One sharing, “Okay, what's next, what is the next area we can work on to get us closer to fulfilling those guidelines completely?”

### Mapping themes to IS theory

To support the interpretation of the themes within IS theory frameworks, we mapped the themes to relevant constructs of NPT^31^ and CFIR^49^ (Table 4). Mapping strengthened our understanding of how teams made change and what influenced uptake of CPGs within the lens of IS theory. Themes like “Navigating Context, Capacity, and Possibility” and “Sizing Up Change” were consistent with constructs of the NPT, including coherence and cognitive participation. In the “Sizing Up Change” and “Plan Phase” themes, we saw that RDNs considered how well recommendations fit within their workflow, which is consistent with compatibility and readiness for implementation of the CFIR model. Themes related to the Plan and Do phases demonstrated the importance of structured tools, PDSA, and using a shared language to support cognitive participation and collective action in alignment with NPT. Feedback and use of data were evident throughout multiple themes and connects to NPT's reflexive monitoring and CFIR's focus on reflection. The use of embedded changes as highlighted in the “Act and Beyond” theme reflects sustainability aspects of CFIR.

**Table 4:** Mapping of themes in comparison to Normalization Process Theory (NPT) and the Consolidated Framework for Implementation Research (CFIR).

Theme	Mapped to NPT constructs	Mapped to CFIR constructs	Rationale	Example from data
Navigating context, capacity, and possibility	Coherence; cognitive participation	Inner setting (structural characteristics, culture, implementation climate); characteristics of individuals (Knowledge and beliefs, self‐efficacy)	Theme emphasizes understanding roles, contextual limitations, and possibility despite of constraints	“We're really good at doing our job without a lot of help… get into our rut of things”
Sizing up change: fresh eyes on familiar practices	Reflexive monitoring; coherence	Intervention characteristics (adaptability, relative advantage); inner setting (compatibility, relative priority); characteristics of individuals (knowledge and beliefs)	Participants reflected on current practices and evaluated changes based on feasibility and context	“What can we actually do, what is realistic… what do we have control over?”
Plan phase: translating shared vision to a	Cognitive participation; collective action	Process (planning, engaging); inner setting (readiness for implementation)	Focuses on building shared understanding, setting goals, and using planning tools to coordinate action. Considers available resources and individuals who can support or champion change	“Doing the actual PDSA worksheets… developing a plan of action to what we already knew was a gap”
Do phase: boots on the ground and eyes to the sky	Collective action; reflexive monitoring	Process (executing and reflecting–evaluating); inner setting (implementation climate, networks and communication)	Theme describes implementation through iterative action and teamwork, balancing patient care with testing	"Adjust and build on what you're already doing…build off that”
Study phase: learning from data and experiences	Reflexive monitoring	Process (reflecting–evaluating); inner setting (learning climate)	Captures structured feedback, data use, and shared learning to assess and adjust practice	“Provided a lot of structure… helped us stay on track and keep things moving forward”
Act phase and beyond: seeing the ripple, strengthening the system	Reflexive monitoring; cognitive participation	Process (reflecting–evaluation and sustainability); inner setting (culture, networks and communication)	Describes reflection on implementation, sustainability, system learning, and intentions to embed changes in future practices	“PDSA cycles… helps you break it down into what's next and who is responsible”

Abbreviations: CFIR, Consolidated Framework for Implementation Research; NPT, Normalization Process Theory; PDSA, Plan–Do–Study–Act.

### Review of process measures and normalization

**Figure 3:**
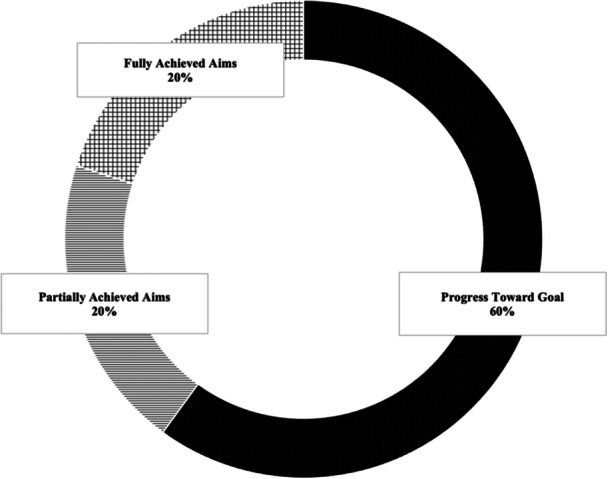
Overview of progress across aims as determined by a manual audit of entries in the GDM Registry Study's quality improvement phase.

A manual audit of registry entries was completed to evaluate achievement of process measures for selected aims. Although not all sites fully met their aims (Figure 3), there were improvements in process measures and meaningful shifts in practices across sites. For example, although site B had an aim to improve the timeliness of MNT from referral to <3 weeks, registry data revealed that initial MNT occurred within 1 week of referral for 75% of their patients in the test cycle period. Site C documented assessment of intention to breastfeed and promotion of breastfeeding benefits for 100% of encounters during the test cycle. Registry data from site D demonstrated that 92% of encounters included technical nutrition education related to comparison of food logs with postprandial readings, which was consistent with their aim to improve self‐monitoring via implementation of a new food intake and blood glucose log. Data from site D demonstrated uptake of counseling related to gestational weight gain, as 91% of encounters in the reference period included nutrition education on the relationship between weight and GDM.

During the closing interviews, RDNs were asked three questions about normalization, referring to whether the recommendation had or would become an embedded or routine practice. Composite scores were calculated for each RDN and the median score across RDNs was 9.00 (95% CI=8.00–10.00). When aggregated at the site level, the median was slightly higher at 9.42 (95% CI=8.00–10.00), suggesting strong implementation (Table 5).

**Table 5:** Composite normalization scores for guideline implementation reported by dietitians in the GDM Registry Study and aggregated by site.

Site	Guideline recommendation	RDN composite^a^	Site composite^b^
Site A	Macronutrients requirements (protein)	10.00	10.00
		10.00	
Site B	Referral for MNT	8.00	8.83
		8.33	
		10.00	
		9.00	
	Prevention of recurrence of GDM or type 2 diabetes	8.00	8.00
		6.33	
		10.00	
		7.67	
Site C	Breastfeeding promotion	10.00	10.00
Site D	Carbohydrate prescription; nutrition assessment—food and nutrition‐related history	9.33	9.17
		9.00	
	IOM weight gain guidelines for pregnancy; nutrition assessment—anthropometrics	4.00	4.00
		4.00	
Site E	Macronutrients requirements (protein)	9.67	9.67
	Physical activity guidance	10.00	10.00

Abbreviations: GDM, gestational diabetes mellitus; IOM, Institute of Medicine; MNT, medical nutrition therapy; RDN, registered dietitian nutritionist.

^a^ The RDN composite score was calculated by averaging responses to three items related to normalization of the guideline/recommendation. RDNs rated how familiar the guideline felt, whether it is currently a normal part of their site's work, and whether it is expected to become a normal part of their site's work. Each item was rated on a 0 (not at all/new) to 10 (completely/familiar) scale. RDN Median 9.00 (95% CI = 8.00–10.00).

^b^ The site composite score was calculated by averaging RDN composite score for each guideline/recommendation implemented. Site median, 9.42 (95% CI = 8.00–10.00).

## DISCUSSION

Our project suggests that PDSA, when paired with other implementation strategies, can support uptake of CPGs into routine care. Assessment of routine practices in comparison with CPGs was a critical step toward implementation. Communication and structured planning tools, such as developing aims and conducting PDSA cycles, were central to driving improvement. Our study illustrates how PDSA cycles can be linked to IS theory; this integration offers a practical, systematic, and adaptable model for future nutrition guideline implementation efforts. Embedding changes into existing workflows and systems helped RDNs stay aligned in their care and may improve broader spread and sustainability. Facilitation and collaboration were key to building capacity and accountability.

The sample of RDNs represented a range of professional experience, with several having limited experience with GDM and QI. At baseline, use of nutrition practice guidelines for GDM was limited and familiarity with NCP was variable. Despite these differences, the overall findings suggest meaningful changes in practice and process improvement. Further, the manual audit of QI entries suggested that care was aligned with CPGs. However, additional analysis is warranted to explore the extent of adherence and changes over the study period. Automated audits using data collected from the ANDHII platform can be used to evaluate guideline implementation.^51^ To support sustained improvement, RDNs could use automated audits within QI processes to provide feedback to clinical teams.

Overall normalization data suggested that the selected guideline recommendations had become a normal part of routine care. However, when normalization was examined by the specific guideline recommendation, we observed that some guidelines, such as prevention of type 2 diabetes and gestational weight gain had lower normalization scores. The findings coincide with interview data and barriers described in the PDSA worksheets. Although not clear, it is conceivable that these recommendations are more complex or influenced by factors beyond the RDN's direct counseling role.

Sites had access to several resources to support implementation, such as training, the QI manual, registry job aide, study portal, and complimentary subscriptions. Dietitians emphasized the value of the QI manual, which offered examples, tools, and worksheets tailored to GDM guidelines. For future studies, RDNs might consider using the Process Improvement Action Worksheets developed by CDR's Quality Management Committee, which offer a comprehensive set of templates and QI resources for dietetics practice.^52^

Dietitians discussed their previous approaches to implementing EBP as largely passive, often limited to continuing education, consulting literature, and reviewing guidelines. These activities did not prompt RDNs to purposefully assess or differentiate practices compared with CPGs, nor did they lead to systematic changes. These experiences correlate with the documented “know‐do” gap that occurs in healthcare. One common perceived barrier was time, as RDNs expressed concerns with their schedules being patient‐focused. Time restraints have been previously identified as a barrier to knowledge translation among RDNs.^53^ Research should quantify the time required for improvement efforts so that schedules and workflows can consistently account for it.

The gap analysis assessment used in this project was simple and yet a critical step toward implementation, as it guided aim development. Defining aims helped sites take ownership of their plans as they prepared for “doing.” Future CPGs should include assessment tools that practitioners can use to evaluate their practices against the CPG standard. In 2023, the Academy published a Guideline Implementation Manual^54^ and more recently a Recommendation Prioritization Tool to help practitioners prioritize implementation.^55^

The PDSA strategy helped sites create a blueprint for implementation. Mapping the qualitative themes to NPT and CFIR highlighted how PDSA helped RDNs operationalize implementation constructs. PDSA supported active planning, problem solving, collaboration, and facilitated feedback. PDSA was accepted by RDNs and offered a flexible yet structured approach for implementation that did not create significant disruptions in their daily workflow. Sites used iterative PDSA cycles to “build” on changes and RDNs expressed intentions for continued improvement. Overall, our study provides a real‐world example of how PDSA can support implementation in nutrition care settings.

Another important aspect of implementation was facilitation, which is consistent with CFIR's construct of Implementation Facilitators.^49^ The study PI worked with each site and brought an outside perspective that proved valuable to implementation. The study PI provided training support, technical assistance, assistance with aim development, refinement of PDSA plans, and problem solving. Monthly check‐ins, reminders related to documentation, and a positive interpersonal relationship were important to creating trust and accountability. Future efforts should consider ways to integrate external facilitation to enhance support for practice change.

### Limitations

The small RDN sample size is a limitation, although inclusion of multiple sites strengthens the study. Some data may be incomplete because of omissions in interviews or worksheets. Although RDN experiences varied, findings suggest implementation strategies were effective.

We did not apply inclusion criteria based on gestational age for patient participants; instead, sites relied on physician referral to define GDM. There is potential for misclassification, particularly in cases of overt diabetes that may not have been appropriately recognized. However, it was not in the scope of the RDN to distinguish those cases.

To avoid biasing registry data entry, an assessment template was initially not used. However, early data showed inconsistent and incomplete assessments, which may confound outcome interpretation. A template was introduced on March 29, 2022, after 13 baseline entries had been submitted by one site; these were retained in the registry.

The unique organization of the registry based on the NCP model and structured terminology facilitates several opportunities to evaluate process measures for each step of the NCP. A primary strength of the study was that sites selected aims that were relevant to their local context and priorities. However, the lack of uniform outcomes limits cross‐site comparisons. Additionally, in hindsight, some aim benchmarks, such as “counsel 100% of patients on…” were unrealistic as not all recommendations are relevant to a patient's individual care plan or goals.

Although the structured registry term list was expansive, it is possible that the registry did not capture all elements of nutrition care that occurred. Dietitians had limited knowledge of the NCPT, especially at baseline. It is possible that they may have omitted data in the registry; however, we did not compare registry data with medical record documentation. Despite this concern, ANDHII incorporates “quality checks” to prompt the practitioner to review data and/or enter missing components related to the NCP.

In the future, we would recommend a third phase of data entry to assess adaptations, sustainment, and fidelity over time. Measuring the degree of adherence before and after would provide concrete evidence of the change in guideline uptake. For example, a previous registry study demonstrated a 3% improvement in guideline adherence because of a basic training.^56^

We did not collect the site gap analysis results, limiting insight into aim selection and contextual factors. However, the use of data triangulation via multiple data collection methods improves the richness of data and our ability to understand experiences.^57^ Further, the comparison of themes to the AI‐derived analyses provided an independent lens to complement but not replace the researcher's analysis. Mapping themes to frameworks such as NPT and CFIR strengthens the interpretation and transferability of our findings that PDSA can be an effective tool for structuring the uptake of CPGs.

It is possible that PDSA alone or as an isolated cycle would not have been as effective without the use of implementation strategies highlighted in this study. However, the study avoided common pitfalls and limitations of PDSA that have been identified in previous literature. For example, a systematic review found that although many projects have reported improvement with PDSA, only a minority achieved specific, measurable goals.^24^ A set of key features for using the PDSA have been published and include iterative cycles, prediction of outcome, small‐scale testing, use of data over time, and documentation.^26^ We were successful in meeting these criteria. Sites completed at least two iterative test cycles (range two to four test cycles) which built on the previous cycle during the QI phase. Sites had to describe their prediction of the outcome in the planning phase of the study. Sites narrowed changes in each PDSA cycle and sample sizes were limited. For example, one site's PDSA worksheet summarized observations from a cycle that included five patients, which is in line with attempts to make small‐scale changes. Data from PDSA worksheets and the registry were collected over time. PDSA was documented at each stage via the submission of aims and PDSA worksheets.

## CONCLUSION

The report describes the application of PDSA as a method for encouraging uptake of CPGs among RDNs. PDSA was accepted by RDNs and fostered a mindset toward iterative improvement. PDSA was strengthened by using implementation strategies, including training and the gap analysis. PDSA helped sites translate knowledge of CPGs into manageable, actionable, and incremental steps. There were several strengths to this study and the findings illustrate the value of using PDSA, alongside tools such as the NCPT and ANDHII, to drive the active work of making CPGs a normal part of routine practice. These methods can be replicated in other settings and with other CPGs.

## AUTHOR CONTRIBUTION

Kerri Lynn Knippen, Lindsay Woodcock, Constantina Papoutsakis, and Diana M. Gonzales‐Pacheco contributed to the conception or design of study. Kerri Lynn Knippen, Lindsay Woodcock, and Constantina Papoutsakis contributed to the data acquisition. Kerri Lynn Knippen contributed to the analysis and interpretation. Kerri Lynn Knippen and Diana M. Gonzales‐Pacheco drafted the initial manuscript and all authors critically revised the manuscript. All authors gave final approval of the version to be published.

## CONFLICT OF INTERESTS STATEMENT

Lindsay Woodcock and Constantina Papoutsakis are employees of the Academy of Nutrition and Dietetics which has a financial interest in the Academy of Nutrition and Dietetics Health Informatics Infrastructure (ANDHII) and Nutrition Care Process Terminology (NCPT) described here. No other potential conflict of interest was reported by the authors.

## Supporting information

Supplemental files are available online at http://ncp.sagepub.com.

Supplemental File 1.

Supplemental File 2.

## References

[1] Braun A , Hill E , Gallo S , et al. Research at the Academy of Nutrition and Dietetics: What, how, and why? J Acad Nutr Diet. 2022;122(11):2150‐2162. 10.1016/j.jand.2022.08.123 35998865

[2] Hoesing H . *Clinical Practice Guidelines: Closing the Gap Between Theory and Practice*. A White Paper by Joint Commission International. 2016.

[3] Hickson M , Papoutsakis C , Madden AM , Smith MA , Whelan K , Academy of Nutrition S . Nature of the evidence base and approaches to guide nutrition interventions for individuals: a position paper from the Academy of Nutrition Sciences. Br J Nutr. 2024;131(10):1754‐1773. 38305040 10.1017/S0007114524000291 PMC11074602

[4] Papoutsakis C , Moloney L , Sinley RC , Acosta A , Handu D , Steiber AL . Academy of Nutrition and Dietetics methodology for developing evidence‐based nutrition practice guidelines. J Acad Nutr Diet. 2017;117(5):794‐804. 27614690 10.1016/j.jand.2016.07.011

[5] Murphy WJ , Hand RK , Abram JK , Papoutsakis C . Impact of diabetes prevention guideline adoption on health outcomes: a pragmatic implementation trial. J Acad Nutr Diet. 2021;121(10):2090‐2100.e1. 33279465 10.1016/j.jand.2020.11.001

[6] Tumilowicz A , Ruel MT , Pelto G , et al. Implementation science in nutrition: concepts and frameworks for an emerging field of science and practice. Curr Dev Nutr. 2018;3(3):nzy080. 10.1093/cdn/nzy080 30864563 PMC6400593

[7] Graham ID , Logan J , Harrison MB , et al. Lost in knowledge translation: time for a map? J Contin Educ Health Prof. 2006;26(1):13‐24. 16557505 10.1002/chp.47

[8] Zhou P , Chen L , Wu Z , et al. The barriers and facilitators for the implementation of clinical practice guidelines in healthcare: an umbrella review of qualitative and quantitative literature. J Clin Epidemiol. 2023 Oct;162:169‐181. 10.1016/j.jclinepi.2023.08.017 37657616

[9] Braithwaite J , Marks D , Taylor N . Harnessing implementation science to improve care quality and patient safety: a systematic review of targeted literature. Int J Qual Health Care. 2014;26(3):321‐329. 10.1093/intqhc/mzu047 24796491

[10] Cochrane LJ , Olson CA , Murray S , Dupuis M , Tooman T , Hayes S . Gaps between knowing and doing: understanding and assessing the barriers to optimal health care. J Contin Educ Health Prof. 2007;27(2):94‐102. 10.1002/chp.106 17576625

[11] Bauer MS , Kirchner J . Implementation science: what is it and why should I care? Psychiatry Res. 2020 Jan;283:112376. 10.1016/j.psychres.2019.04.025 31036287

[12] Woteki CE , Colón F . A vision for advancing nutrition science in the United States. Nutr Today. 2025;60(1):6‐9. 10.1097/NT.0000000000000732

[13] Heitman K , Hubbard J , Easter L , Kilkus J . Looking to the future: agendas, directions, and resources for nutrition research. Nutr Clin Pract. 2024;39(4):772‐782. 10.1002/ncp.11154 38667339

[14] Bauer MS , Damschroder L , Hagedorn H , Smith J , Kilbourne AM . An introduction to implementation science for the non‐specialist. BMC Psychol. 2015;3(1):32‐39. 10.1186/s40359-015-0089-9 26376626 PMC4573926

[15] Brown M , Rosenthal M , Yeh DD . Implementation science and nutrition: from research to practice. Nutr Clin Pract. 2021;36(3):586‐597. 10.1002/ncp.10677 34021636

[16] Vergili JM , Proaño GV , Jimenez EY , Moloney L , Papoutsakis C , Steiber A . Academy of nutrition and dietetics commentary on the phosphorus recommendation in the KDOQI clinical practice guidelines for nutrition in CKD: 2020 update. J Ren Nutr. 2024;34(3):192‐199. 38007185 10.1053/j.jrn.2023.11.001

[17] Tyler A , Glasgow RE . Implementing improvements: opportunities to integrate quality improvement and implementation science. Hosp Pediatr. 2021;11(5):536‐545. 10.1542/hpeds.2020-002246 33910971 PMC8074111

[18] Kaplan HC , Walsh KE . Context in implementation science. Pediatrics. 2022;149(suppl 3):e2020045948C. 10.1542/peds.2020-045948C 35230429

[19] Leeman J , Rohweder C , Lee M , et al. Aligning implementation science with improvement practice: a call to action. Implement Sci Commun. 2021;2(1):99. 10.1186/s43058-021-00201-1 34496978 PMC8424169

[20] Bierbaum M , Best S , Williams S , et al. The integration of quality improvement and implementation science methods and frameworks in healthcare: a systematic review. BMC Health Serv Res. 2025;25(1):558‐559. 10.1186/s12913-025-12730-9 40241054 PMC12001488

[21] Malone S , Newland J , Kudchadkar SR , et al. Sustainability in pediatric hospitals: an exploration at the intersection of quality improvement and implementation science. Front Health Serv. 2022 Nov 10;2:1005802. 10.3389/frhs.2022.1005802 36925889 PMC10012775

[22] Langley GJ , Moen RD , Nolan KM , Nolan TW , Norman CL , Provost LP . The Improvement Guide: A Practical Approach to Enhancing Organizational Performance. 2nd ed. Jossey‐Bass; 2009.

[23] Reed JE , Card AJ . The problem with plan‐do‐study‐act cycles. BMJ Qual Saf. 2016;25(3):147‐152. 10.1136/bmjqs-2015-005076 PMC4789701 26700542

[24] Knudsen SV , Laursen HVB , Johnsen SP , Bartels PD , Ehlers LH , Mainz J . Can quality improvement improve the quality of care? A systematic review of reported effects and methodological rigor in plan‐do‐study‐act projects. BMC Health Serv Res. 2019;19(1):683. 10.1186/s12913-019-4482-6 31585540 PMC6778385

[25] Knippen K , Woodcock L , Papoutsakis C . From research to practice: putting guidelines into action for gestational diabetes. J Acad Nutr Diet. 2023;123(10):A33. 10.1016/j.jand.2023.08.094

[26] Taylor MJ , McNicholas C , Nicolay C , Darzi A , Bell D , Reed JE . Systematic review of the application of the plan‐do‐study‐act method to improve quality in healthcare. BMJ Qual Saf. 2014;23(4):290‐298. 10.1136/bmjqs-2013-001862 PMC3963536 24025320

[27] O'Brien BC , Harris IB , Beckman TJ , Reed DA , Cook DA . Standards for reporting qualitative research: a synthesis of recommendations. Acad Med. 2014;89(9):1245‐1251. 24979285 10.1097/ACM.0000000000000388

[28] The Academy of Nutrition and Dietetics . *Nutrition Terminology Reference Manual (eNCPT): Dietetics Language for Nutrition Care*. *Electronic Nutrition Care Process Terminology (eNCPT)* . https://www.ncpro.org/

[29] Bechtold ML , Matteson‐Kome ML . Implementation science using the Plan‐Do‐Study‐Act (PDSA) cycle: addressing hospital malnutrition with the global malnutrition composite score. Nutr Clin Pract. 2025;1‐10. doi:10.1002/ncp.70041 10.1002/ncp.70041 41015886

[30] Academy of Nutrition and Dietetics . Evidence Analysis Library . https://www.andeal.org/

[31] May CR , Mair F , Finch T , et al. Development of a theory of implementation and integration: normalization process theory. Implement Sci. 2009;4(1):29. 19460163 10.1186/1748-5908-4-29 PMC2693517

[32] Institute for Healthcare Improvement . Aim Statement Worksheet . https://www.ihi.org/resources/tools/aim-statement-worksheet

[33] Duarte‐Gardea MO , Gonzales‐Pacheco DM , Reader DM , et al. Academy of Nutrition and Dietetics gestational diabetes evidence‐based nutrition practice guideline. J Acad Nutr Diet. 2018;118(9):1719‐1742. 29859757 10.1016/j.jand.2018.03.014

[34] Franz MJ , Boucher JL , Green‐Pastors J , Powers MA . Evidence‐based nutrition practice guidelines for diabetes and scope and standards of practice. J Am Diet Assoc. 2008;108(4):S52‐S58. 18358257 10.1016/j.jada.2008.01.021

[35] American Diabetes Association Professional Practice Committee, American Diabetes Association Professional Practice Committee: 15 . Management of diabetes in pregnancy: standards of medical care in Diabetes—2022. Diabetes Care. 2022;45(suppl 1):S232‐S243. 34964864 10.2337/dc22-S015

[36] Institute of Medicine (US) and National Research Council (US) . *Committee to Reexamine IOM Pregnancy Weight Guidelines*. 2009.

[37] Institute for Healthcare Improvement . Quality improvement essentials toolkit. 2017. https://www.ihi.org/resources/tools/quality-improvement-essentials-toolkit.

[38] Swan WI , Pertel DG , Hotson B , et al. Nutrition care process (NCP) update part 2: developing and using the NCP terminology to demonstrate efficacy of nutrition care and related outcomes. J Acad Nutr Diet. 2019;119(5):840‐855. 30660633 10.1016/j.jand.2018.10.025

[39] Colin CR , Woodcock L , Wright LY , Yakes Jimenez E , Papoutsakis C . The need for and challenges of nutrition and dietetics registry studies: an update on the academy of nutrition and dietetics health informatics infrastructure. J Acad Nutr Diet. 2023;123(4):673‐682. 36623691 10.1016/j.jand.2023.01.002

[40] Swan WI , Vivanti A , Hakel‐Smith NA , et al. Nutrition care process and model update: toward realizing people‐centered care and outcomes management. J Acad Nutr Diet. 2017;117(12):2003‐2014. 28988837 10.1016/j.jand.2017.07.015

[41] Chui TK , Proaño GV , Raynor HA , Papoutsakis C . A nutrition care process audit of the national quality improvement dataset: supporting the improvement of data quality using the ANDHII platform. J Acad Nutr Diet. 2020;120(7):1238‐1248.e1. 10.1016/j.jand.2019.08.174 31668603

[42] Colin C , Arikawa A , Lewis S , et al. Documentation of the evidence‐diagnosis link predicts nutrition diagnosis resolution in the academy of nutrition and dietetics' diabetes mellitus registry study: a secondary analysis of nutrition care process outcomes. Front Nutr. 2023 Mar 9;10:1011958. 10.3389/fnut.2023.1011958 36969819 PMC10034103

[43] Braun V , Clarke V . Using thematic analysis in psychology. Qual Res Psychol. 2006;3(2):77‐101.

[44] Ioannou E , Humphreys H , Homer C , Purvis A . Barriers and system improvements for physical activity promotion after gestational diabetes: a qualitative exploration of the views of healthcare professionals. Diabetic Med. 2024;41(12):1‐11. 10.1111/dme.15426 39153179

[45] Liapi F , Chater AM , Kenny T , Anderson J , Randhawa G , Pappas Y . Implementation of step‐down intermediate care (IC) in Buckinghamshire, UK: a qualitative evaluation study of healthcare professionals' experiences and perspectives. Health Soc Care Commun. 2024;30(2):1‐11. 10.1155/2024/8864478

[46] Kelly Y , O'Rourke N , Flynn R , Hegarty J , Keyes LM . Exploring the implementation of national health and social care standards in Ireland: a qualitative descriptive study. J Adv Nurs. 2025;81(3):1489‐1504. 10.1111/jan.16346 39046217 PMC11810485

[47] OpenAI . ChatGPT [large language model]. ChatGPT‐4. Updated 2023.

[48] AILYZE . 1 AILYZE. www.ailyze.com

[49] Damschroder LJ , Aron DC , Keith RE , Kirsh SR , Alexander JA , Lowery JC . Fostering implementation of health services research findings into practice: a consolidated framework for advancing implementation science. Implement Sci. 2009;4(1):50. 19664226 10.1186/1748-5908-4-50 PMC2736161

[50] Sivakumar B , Mak J , Bafagih S , Arcand J . Implementation science in nutrition practice: a review of the Consolidated Framework for Implementation Research. Nutr Clin Pract. 2025;1‐25. doi:10.1002/ncp.70020 10.1002/ncp.70020 PMC12590315 40886064

[51] Lamers‐Johnson E , Kelley K , Sánchez DM , et al. Academy of Nutrition and Dietetics nutrition research network: validation of a novel nutrition informatics tool to assess agreement between documented nutrition care and evidence‐based recommendations. J Acad Nutr Diet. 2022;122(4):862‐872. 10.1016/j.jand.2021.03.013 33903080

[52] Commission on Dietetic Registration, Quality Management Committee . Quality, Standards, and Interoperability Team Process Improvement Action Plan Worksheet . 2024. Accessed April 14, 2025. https://www.cdrnet.org/vault/2459/web//Process%20Improvement%20How%20To%20Guide%20-%20UPDATED.pdf

[53] Soguel L , Vaucher C , Bengough T , Burnand B , Desroches S . Knowledge translation and evidence‐based practice: a qualitative study on clinical dietitians' perceptions and practices in Switzerland. J Acad Nutr Diet. 2019;119(11):1882‐1889. 31296425 10.1016/j.jand.2019.04.017

[54] Council on Research 2022‐23 Implementation Subcommittee . Implementing Evidence: From Guidelines to Daily Practice . Evidence Analysis Center, Academy of Nutrition and Dietetics. 2023. Accessed April 14, 2025. https://www.andeal.org/guideline-implementation

[55] Council on Research 2023‐24 Implementation Subcommittee, Evidence Analysis Center . Recommendation Prioritization Tool . Academy of Nutrition and Dietetics. 2024. Accessed April 14, 2025. https://www.andeal.org/guideline-implementation

[56] Murphy WJ , Hand RK , Abram JK , Papoutsakis C . Impact of diabetes prevention guideline adoption on health outcomes: a pragmatic implementation trial. J Acad Nutr Diet. 2021;121(10):2090‐2100.e1. 33279465 10.1016/j.jand.2020.11.001

[57] Moon MD . Triangulation: a method to increase validity, reliability, and legitimation in clinical research. J Emerg Nurs. 2019;45(1):103‐105. 10.1016/j.jen.2018.11.004 30616761

