# Supplementary material for: Implementation of clinical practice guidelines using the Plan–Do–Study–Act framework: The methodology and experiences of the Academy of Nutrition and Dietetics Health Informatics Infrastructure Registry Study on gestational diabetes mellitus
Source: Nutr Clin Pract. 2025 Oct 2;40(6):1465–82. doi: 10.1002/ncp.70043 (PMC12590319; doi:10.1002/ncp.70043)
Supplement: Supplementary file 1 — Supplemental File 1. [file NCP-40-1465-s002.pdf]

GUIDELINE IMPLEMENTATION

& QUALITY IMPROVEMENT WORKBOOK

THE GDM REGISTRY STUDY

How to use this Workbook

This workbook was developed for the GDM Registry Study. Dietitians (RDNs) and sites participating in this study should use this workbook to develop your quality improvement (QI) project focused on evidence-based guidelines and GDM. This workbook will provide an overview of implementation and QI principles and how these strategies can improve guideline uptake and quality of care.

Dietitians will want to complete the required GDM evidence-based guideline midpoint training activities. In addition, please carefully review relevant guideline recommendations from the Academy of Nutrition and Dietetics (the Academy) and the American Diabetes Association (ADA). Collectively these resources, will help you assess your current practices and identify gaps in care where evidence-based practice may lead to improved outcomes.

This workbook includes materials related to QI and guideline implementation. The workbook includes customizable tools, worksheets, examples, and resources you can use to develop your specific project. We recommend that you first complete the required online training before moving into the workbook activities. You may find that keeping notes in this workbook will help you reflect during the training. You will want to save your materials so you can monitor your progress with the project.

Introduction

QI focuses on systematic and ongoing work to standardize processes leading to improvement in outcomes [1] The Institute of Medicine (IOM)[2] has defined six dimensions of quality (Figure 1). There are a variety of QI methods that can be used in healthcare. The remainder of this workbook will focus on the Model for Improvement and PDSA- Plan Do Study Act cycles for QI.

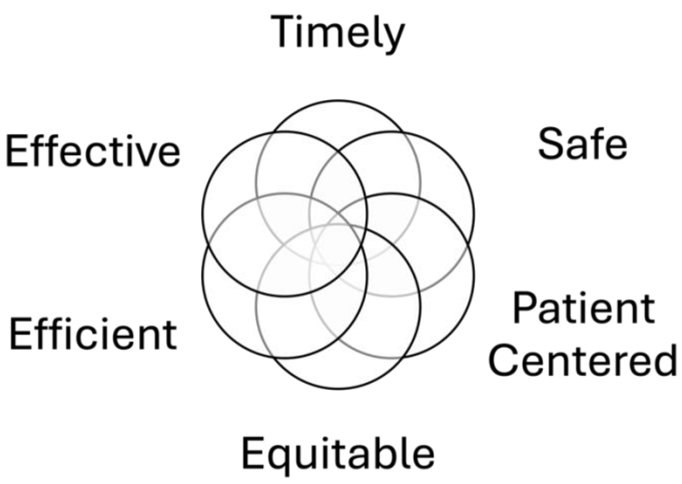

Figure 1. Dimensions of Quality

[1] Commission on Dietetic Registration. *Quality Management*. Website: https://www.cdrnet.org/quality
[2] Institute of Medicine (US) Committee on Quality of Health Care in America. *Crossing the Quality Chasm: A New Health System for the 21st Century*. Washington, DC: National Academies Press; 2001.

QI Methods

QI methods are systematic and change-action oriented. QI can be used as a method of "implementation" to increase uptake of clinical practice guidelines, such as the Academy's Evidence-Based Nutrition Practice Guidelines (EBNPG) for GDM. GDM Registry Study Sites will use the Model for Improvement which includes 2 main components (1) 3-key questions and (2) Plan Do Study Act (PDSA) Cycles.[3] There are four main steps in each PDSA cycle (Figure 2). Sites will use the PDSA cycle method to make iterative, small-scale changes to their clinical practice for GDM.

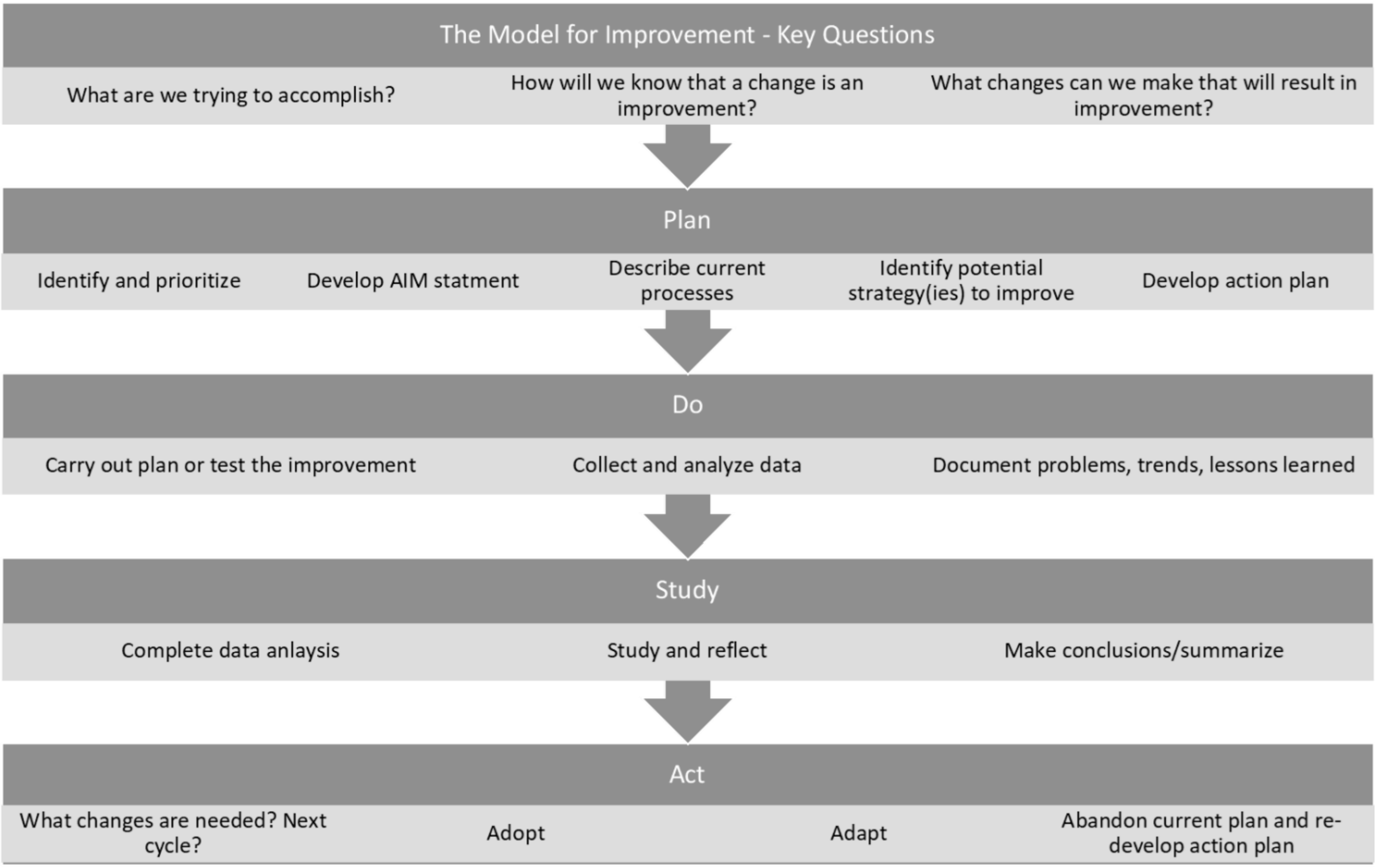

Figure 2. Plan, Do, Study, Act Processes based on Deming's Model for Improvement[4]

PDSA cycles are selected for this project because they allow for rapid evaluation of the "change" improvement. Testing can help us understand which changes lead to the desired improvement outcome. Testing can also help sites determine whether the change(s) work in the clinical environment, the possible costs, and side effects of change.

---

[3] Institute for Healthcare Improvement. IHI website. https://www.ihi.org/library/model-for-improvement
[4] Langley GJ, Moen RD, Nolan KM, Nolan TW, Norman CL, Provost LP. The Improvement Guide: A Practical Approach to Enhancing Organizational Performance 2009.

ASSESS PRACTICES: After completing the baseline phase and midpoint training, RDNs at each site should reflect on the current nutrition counseling practices related to GDM. Sites should use the **Assessment Worksheets** in this workbook to reflect on current practices and gaps in care related to GDM. This assessment process may help sites identify the focus of their QI aims.

FORM YOUR TEAM: Before getting started with the planning phase, each site should identify their Team. The team may consist of other RDNs, clinical nutrition manager, patients, medical assistants, physician leader, midwife, diabetes education staff, nursing, etc. The team should identify an RDN "champion" for their site's QI plan. Your team can include those external to your nutrition practice, if applicable.

PLAN: The first step "Plan" consists of setting aims, making predictions, and creating a plan for change and data collection. **Each site will identify 2 aims for the QI phase of the study**. The aims must be related to evidence-based practice guidelines. We encourage RDNs to reflect on the baseline period and the evidence-based nutrition practice guidelines for GDM for this phase of the study. Sites should examine their clinical workflow and overall nutrition care process using the **Assessment Worksheet.**

Once a site has identified the general objectives (sometimes called global aim) for their QI work, they will create a SMART aim statement. The workbook provides additional tools you can use to create your aim statement. Along with creating the aim statement, RDNs will need to establish measures or identify how they will know if a change results in improvement. **Measures should be able to be documented in the GDM Registry (ANDHII). Sample measures and strategies for creating a data collection plan are shared in this workbook.** Sites will need to identify the change(s) they wish to implement and create an action plan.

DO: During the "Do" phase sites will implement the action plan and test the change(s). Sites should document problems, observations during the do phase. Sites will submit a worksheet for each PDSA cycle to report their progress and observations. We recommend 1-2 PDSA cycles per month.

STUDY: Sites will need to analyze and study the data they have collected for each change. Sites will compare their data to their predicted outcomes and reflect on what was learned.

ACT: During the last step of the PDSA cycle, sites will identify what was learned and use that information to inform the plan. This information may help sites refine changes, identify modifications, or prepare a plan for the next PDSA cycle.

QI PHASE SITE REQUIREMENTS

- ☐ RDNs at each site should complete the midpoint online required training before moving forward.
- ☐ Sites will complete the **guideline assessment** found in this manual. This assessment process includes a review of the team, roles, expectations, resources, and current standards of care related to GDM and nutrition care. Sites will complete this assessment collectively.
- ☐ Sites will form their team and identify a "RDN Site Champion" for their QI phase. Consider who you may want to engage in your team (other staff, clinicians, patients, etc.).
- ☐ Sites will choose 2 aims for the QI phase – **aims must be related to evidence-based practice related to GDM.**
  - **Aims should focus on outcomes that can be documented in the registry (ANDHII).**
  - To create an aim, sites should reference the Evidence Analysis Library, the Evidence Based Nutrition Practice Guidelines for GDM, and other standards of clinical care for GDM, such as the ADA Standards of Clinical Care.
  - Aims must be approved by the principal investigator before moving forward with planning.
- ☐ **Sites** will engage in iterative PDSA cycles. Each site will be required to complete at least 2 PDSA test cycles. We recommend 1 to 2 PDSA cycles per month during the QI Phase.
- ☐ Sites will complete a PDSA planning sheet before starting a PDSA test cycle. This will be submitted to the principal investigator for feedback and/or approval.
- ☐ After a test cycle, sites will submit a PDSA worksheet to describe changes, improvements, barriers that occurred.
- ☐ Sites will be expected to document approximately 30 registry encounters during the QI phase.

For more information on the Model for Improvement, please visit the Institute for Healthcare Improvement's (IHI) website.[5] Trainings, worksheets, videos and toolkits are available at IHI's website.

RDNs can use this workbook to help with the planning process. Required worksheets and optional tools are linked in this manual. An example of a PDSA cycle is shared on the next page. The aim is focused on weight gain guidance with GDM.

---

[5] Institute for Healthcare Improvement. IHI website. https://www.ihi.org/

Sample PDSA Cycle: Pregnancy Weight Change Recommendations

Aim: By the end of the QI period, 60% of GDM clients will achieve the recommended pregnancy weight change based on the Institute of Medicine's 2009 guidelines.

PLAN: ***Plan for change and data collection.***

Plan for change

- ☐ RDNs will receive training on the IOM recommendations
- ☐ Nutrition assessment will include review of pre-pregnancy weight, determination of pre-pregnancy BMI, and current pregnancy weight change
- ☐ RDNs will assess current weight gain based on # weeks gestation
- ☐ RDNs will provide nutrition prescription (energy prescription) that addresses weight change recommendations in addition to other factors (e.g., physical activity, # week gestation, etc.)
- ☐ RDNs will follow up with clients and re-assess weight change with subsequent MNT encounters
- ☐ RDNs will adjust nutrition prescription as needed based on weight change while also considering glycemic control and other related labs, procedures, etc.

Plan for data collection

- ☐ RDNs will review notes/registry reports to determine if nutrition assessment addresses review of pre-pregnancy weight, pre-pregnancy BMI, and current pregnancy weight change
- ☐ RDNs will assess sample of notes/ANDHII reports to determine if the nutrition prescription and/or nutrition intervention documents weight gain guidance or an individualized energy prescription based on weight change guidance based on the IOM recommendation

DO: ***Carry out the change, collect data, and begin analysis.***

- ☐ RDNs will determine pre-pregnancy weight and use IOM guidance to counsel on weight gain recommendations in pregnancy
- ☐ RDNs will plot weight change in comparison to recommended weight change using standard weight gain charts
- ☐ RDNs will review notes of GDM clients referred for MNT in the past XXX period of time (e.g., 1-week, 2-weeks) to determine if the guideline is being implemented

STUDY: ***Complete analysis of data and debrief.***

Examples of discussion/reflection points for the "study phase" shared:

- ☐ Review data and discuss whether patients are being counseled on weight gain guidance and if weight change recommendations are considered into nutrition prescription.
- ☐ Discuss if there are other measures to support this change? Would an automatic flag in your electronic medical record to assess pre-pregnancy BMI trigger counseling? Would a checklist for RDNs prompt counseling? Does plotting the weight change on a standard pregnancy weight gain chart improve counseling practices?
- ☐ As you study the "change" review how closely your results match the improvement set out to achieve based on your objective. Did you meet your benchmark?
- ☐ As you review the cycle, discuss what was learned and consider how this can improve your next cycle.

ACT: ***List actions to take for next cycle. Repeat, adapt, adopt the change, or scale up, or abandon change.***

- ☐ Repeat this test for another XX period of time (e.g., # days, 1-week, 2-weeks).
- ☐ Adapt change and run a second PDSA cycle after initiating a checklist that addresses weight gain guidance for GDM clients.

Planning for Change: Assessing Current Practices Worksheet

Complete the following worksheet to identify possible gaps in care, workflow, or delivery of services.

Discuss your results as a team before planning for change. Save this worksheet for your team's QI planning. This does not need submitted for the study. This will help your team reflect on where you started and QI strategies to focus on.

Setting Description

Select all that apply:

☐ General outpatient

☐ Diabetes self-management program

☐ Specialty practice (e.g., endocrinology, maternal-fetal medicine)

☐ Other: ______________________________

Who do you Serve

Describe your patient population relative to gestational diabetes, e.g., average age, demographic characteristics, insurance access/coverage, etc.

____

____

____

____

MNT Encounters Frequency

Average # of MNT visits for GDM during pregnancy: ____________Average # of MNT visits for GDM during postpartum: ____________

Encounter Types

Select all that apply:

☐ Group Education

☐ Individual (assessment, counseling, education, re-assessment)

☐ Shared medical appointment

☐ In-person

☐ Telephonic patient communication

☐ Telehealth (real-time audio + video)

Team and Roles

of RDNs who see patients with GDM: ____________# of other staff members: ____________

Who is on your team for GDM-related care? (Include titles and roles):

____

____

____

____

Who is the lead for GDM management: ____

Processes and Protocols

Are there any processes/protocols/standing orders in place for managing patients? ☐ Yes ☐ No

Describe current roles, standards, and expectations (e.g., clinical workflow, medication titration, communication):

____

____

____

____

Communication

Access to full medical record (paper or EMR): ☐ Yes ☐ No Able to communicate with other team members: ☐ Yes ☐ No

Describe your referral system and communication processes:

____

____

____

Barriers to Care

____

____

____

Notes or Observations

____

____

Assessment of Current Evidence-Based Nutrition Guideline Practices for GDM[6]

**Recommendation/Guideline**	**How often is this routine practice?** ***Always, Sometimes, Never***	**Who is responsible?** ***(RDN, other: describe)***	**Resources** ***(available or needed)***
NUTRITION ASSESSMENT & RE-ASSESSMENT			
Food-nutrient intake and related history – calorie intake			
Food-nutrient intake and related history – serving sizes			
Food-nutrient intake and related history –type and amount of carbohydrate (fiber)			
Food-nutrient intake and related history –type and amount of protein			
Food-nutrient intake and related history –type and amount of fat			
Meal and snack patterns (frequency, duration)			
Intake of high calorie, low nutrient dense foods/beverages			
Food preferences, avoidances, intolerances, allergies			
Appetite and changes in appetite			
Eating environment (e.g., meals away from home)			
Diet history/behavior (e.g., previous diets, adherence, disordered eating)			

[6] Guideline recommendations related to the nutrition care process were based on Duarte-Gardea MO, Gonzales-Pacheco DM, Reader DM, et al. Academy of Nutrition and Dietetics Gestational Diabetes Evidence-Based Nutrition Practice Guideline. *J Acad Nutr Diet*. 2018;118(9):1719-1742. doi:10.1016/j.jand.2018.03.014

Food preparation, food safety			
Pharmacologic therapy			
Substance use			
Use of dietary supplements			
Knowledge, beliefs, attitudes			
Physical activity and function			
Anthropometric measures (e.g., height, weight, pre-pregnancy weight and BMI, weight change in pregnancy)			
Biochemical data (e.g., glucose challenge test, OGTT, A1c, fasting glucose, self-monitoring glucose data, urinary ketones)			
Medical tests and procedures (e.g., ultrasounds, BPP, non-stress testing)			
Thyroid function			
Micronutrient levels if applicable (e.g., vitamin D)			
Kidney function			
Nutrition Focused Physical Exam Findings			
Client History – age			
Client History – single or multiples			
Client history – # weeks gestation			
Client history – Risk factors for GDM			
Client history – Family history of DM			

Client history – General health (e.g., vital signs)			
Client history –other medical history			
Client history – GI symptoms (e.g., nausea, vomiting, diarrhea, constipation, heart burn)			
Health literacy and numeracy			
Education and occupation			
Social history (e.g., social support)			
NUTRITION INTERVENTION			
Educate on physiology of GDM			
Energy prescription			
Weight gain in pregnancy			
Carbohydrate prescription			
Macronutrient requirements (carbohydrate, protein, fat)			
Use of high intensity sweeteners			
Breakfast and postprandial hyperglycemia			
Physical activity recommendations with pregnancy			
Micronutrient supplementation			
Distribution of meals and snacks			
Dietary patterns (e.g., low glycemic index, DASH, Mediterranean)			
Alcohol abstinence counseling			
Self-monitoring of glucose			

Pharmacological therapy			
MNT frequency and duration (minimum of 3 visits)			
Risk for type 2			
Postpartum screening recommendations for type 2			
Lifelong screening recommendations for type 2			
Promotion of breastfeeding			
Postpartum body weight			
Lifestyle modification for diabetes prevention			
OTHER CONSIDERATIONS: COORDINATION AND COLLABORATION OF CARE			
Process for postpartum MNT			
Process to communicate with clinical care team			
Process to hand off patient to referring provider or PCP			
Process to check if patients complete postpartum visit			
Process for scheduling postpartum diabetes screening			
Process to remind patient of postpartum diabetes screening			
Process to communicate test results to patients			
Process to follow up with patients who miss postpartum visit/screening			
Process to refer to community resources, (e.g., DPP or DSME when appropriate)			

Developing Aims for the GDM Registry Study QI Project

Sites will select <u>2 aims</u> (e.g., goals) for their QI project. The 2 aims selected by a site must reflect uptake of evidence-based clinical practice or evidence-based nutrition practice guidelines. Sites should review the Academy of Nutrition and Dietetics Evidence-Based Nutrition Practice Guidelines and ADA's Standards of Clinical Care to identify a potential focus or aim for the GDM QI project. These materials are shared on the study portal.

Aims must be reviewed by the study PI before you can move forward with the QI phase.

General guidance on writing aims:

- ✓ **Write it clearly:** Describe exactly what you want to improve in one concise sentence.
- ✓ **Make it measurable:** Decide how you will know your aim is achieved.
- ✓ **Define your target:** Specify who or what will be impacted.
- ✓ **Set a realistic goal:** Choose a benchmark that is achievable.
- ✓ **Pick a timeline:** Determine when you want to see the change happen.

Please refer to the IHI website to access the Aim worksheet.[7]

Sites must submit one worksheet for each aim.

[7] Institute for Healthcare Improvement. *Aim Statement Worksheet*. IHI Website. https://www.ihi.org/library/tools/aim-statement-worksheet

Driver diagrams can be used to describe the "theory or logic" of how processes might be better or how you can refine, develop, or implement the nutrition care plan to achieve your aim. Drivers are components or points of the system that can lead to achievement of your aim.[8] Drivers are sometimes categorized as primary or secondary. We have classified drivers as "key drivers" below. Drivers must be measurable, and most will relate to process outcomes. **The nutrition care process model and recommendations from the evidence-based nutrition practice guidelines can be used as leverage to reach aims.** Sites are encouraged to think about "key drivers" to identify change ideas.

Examples of Key Drivers

- ☐ Knowledge of RDNs
- ☐ Nutrition care process
- ☐ Evidence based nutrition practice guidelines
- ☐ Standards of care
- ☐ Communication among team members
- ☐ Scheduling Process
- ☐ Clinical workflow
- ☐ Time
- ☐ Resources
- ☐ Variation in nutrition care
- ☐ Patient experience
- ☐ Care pathways
- ☐ Access to services
- ☐ Food environment of patients
- ☐ Social support of patients
- ☐ Coordination of care
- ☐ Transitions of care
- ☐ Patient perceptions, awareness, knowledge, attitudes, beliefs
- ☐ Scope of practice for RDNs
- ☐ Self-management support
- ☐ Electronic Medical Records
- ☐ Registry or Information Systems

Want to create a driver diagram to visualize what may impact or lead to your intended outcome?

Visit the Institute for Healthcare Improvement's website for examples and a free template.[8]

Examples of Change Ideas

- ☐ Work with staff to understand and optimize referral procedures
- ☐ RDN training on nutrition care process
- ☐ Standardized process to assess pre-pregnancy BMI with initial MNT
- ☐ RDN counseling on the IOM recommendations
- ☐ Standardizing MNT scheduling
- ☐ Delineate roles for care coordination in a multi-disciplinary team
- ☐ Standard template tool to provide individualized calorie nutrition prescription to achieve weight and glycemic goals
- ☐ RDNs provide evidence based physical activity assessment and guidance
- ☐ Process to monitor SMBG and patient generated data
- ☐ RDN counseling on breakfast and post prandial hyperglycemia
- ☐ RDN counseling on relationship of carbohydrate to SMBG
- ☐ RDN relationships with lactation consultants
- ☐ Standardized process for RDN counseling on the risk of future GDM and type 2 DM
- ☐ Develop system for referral to community resources
- ☐ Implement an OGTT reminder system

[8] Institute for Healthcare Improvement. *Driver Diagram*. IHI Website. https://www.ihi.org/library/tools/driver-diagram

BRAINSTORMING CHANGE*:* Use the worksheet below to link your aim statement to key drivers, outcomes, and changes. Potential improvements could include but are not limited to: improved food-nutrient intake, increased physical activity, achievement of maternal blood glucose targets, achievement of maternal weight gain target, prevention of adverse maternal or fetal outcomes. Last, evaluate the change concepts you have identified as having potential to have low or high impact and rate how easy or hard the change is to implement.

Aim Statement	How will you know if change is an improvement (e.g., possible outcomes)?	Key Drivers	What change ideas can result in improvement?	Is the change likely to have low or high impact?	Is the change easy or hard to implement?

ASSESSING AND PRIORITIZING THE GUIDELINE

Sites are encouraged to think about the feasibility of guideline implementation by using the questions shared below. Mark the response that describes your site's disagreement/agreement with each statement. Discuss results with your team.

	Strongly disagree	Disagree	Agree	Strongly agree
We can easily integrate the [guideline/recommendation/intervention] into existing work/clinical flow.				
The [guideline/recommendation/intervention] disrupts working relationships.				
Sufficient training is available to help staff implement the [guideline/recommendation/intervention].				
Sufficient resources are available to support the implementation of the [guideline/recommendation/intervention].				
Management/decision makers adequately support the [guideline/recommendation/intervention].				
The team agrees that the [guideline/recommendation/intervention] is worthwhile.				
The team can see how the [guideline/recommendation/intervention] differs from usual ways of working.				
The team and site staff have a shared understanding of the purpose of the [guideline/recommendation/intervention].				

Adapted from the NoMad Tool[9]

[9] Finch, T.L., Girling, M., May, C.R., Mair, F.S., Murray, E., Treweek, S., Steen, I.N., McColl, E.M., Dickinson, C., Rapley, T. (2015). NoMad: Implementation measure based on Normalization Process Theory. [Measurement instrument]. Retrieved from http://www.normalizationprocess.org.

Reflection on Normalization

We recommend that you routinely reflect on normalization of the guideline. You can do this at the start /end of any new PDSA cycle. "Guideline/recommendation/intervention" refers to evidence-based practice guidelines, research, or standards of care. Questions adapted from the NoMad tool.[10]

**List the evidence based GDM guideline(s)/recommendation(s)/intervention(s) your site is interested in or focused on currently? This should relate to your QI Aims.**

When you/your site uses the [guideline/recommendation/intervention], how familiar does it feel?

Still feels very new — Feels completely familiar

0 1 2 3 4 5 6 7 8 9 10

***Reflect on why you answered the way you did. Why might that be?**

Do you feel the [guideline/recommendation/intervention] is currently a normal part of your site's work?

Not at all — Somewhat — Completely

0 1 2 3 4 5 6 7 8 9 10

***Reflect on why you answered the way you did. Why might that be?**

Do you feel the [guideline/recommendation/intervention] will become a normal part of your site's work?

Not at all — Somewhat — Completely

0 1 2 3 4 5 6 7 8 9 10

***Reflect on why you answered the way you did. Why might that be?**

---

[10] Adapted from: Finch, T.L., Girling, M., May, C.R., Mair, F.S., Murray, E., Treweek, S., Steen, I.N., McColl, E.M., Dickinson, C., Rapley, T. (2015). NoMad: Implementation measure based on Normalization Process Theory. [Measurement instrument]. Retrieved from http://www.normalizationprocess.org.

Creating a Data Collection Plan

Keep in mind the nature of PDSA cycles – rapid cycles for change improvement. Sites should develop a data collection plan that is feasible, timely, and can lead to information to inform the next cycle. The focus is on small tests which can lead to significant gains/changes over time for improvement. There are different types of QI outcomes that may be monitored in a data collection plan. For more information about types of outcomes (process, clinical, psychosocial, patient reported, or patient generated) please see Standard 6 of the 2022 National Standards for Diabetes Self-Management Program.[11] Examples of indicators for various types of outcomes are shared below.

Process Outcome Indicators

- Attendance; Timing of Appointments; Number of Visits; % patients where "x" was assessed as reflected by registry report; % patients who received "x" intervention as reflected by registry report

Clinical Outcome Indicators

- % self monitoring glucose, fasting in range; % self monitoring glucose, postprandial in range; Pregnancy-maternal-fetal outcomes (BPP, growth ultrasound, delivery type); Weight change

Behavioral Outcome Indicators

- Medication adherence; Breastfeeding; Self monitoring behavior

Patient Reported Outcome Indicators

- Self-efficacy; Perceived risk; Food-nutrition related knowledge

Patient Generated Data Indicators

- Blood glucose patterns determined by glucometer download/log; Weight; Physical Activity determined by wearable technology

IMPORTANT – Sites must identify outcomes that can be documented in the registry. If you are not sure if an outcome can be documented after consulting the NCP Terminology, please contact the study PI. Sites may choose (optional) to collect data or feedback outside the registry-de-identified results can be shared in aggregate on the PDSA worksheet.

The following page also includes examples of how sites can use the Nutrition Care Process to develop their data collection plan.

[11] American Diabetes Association. *2022 National Standards for Diabetes Self-Management Education and Support: Standard 6. Measuring and Demonstrating Outcomes of DSMES Services*. 2022.

The Nutrition Care Process Model can be a helpful tool for determining outcomes related to the uptake of evidence-based nutrition practice guidelines for GDM. Examples of process outcomes are shared below in alignment with the nutrition practice guidelines for GDM.

- *NUTRITION ASSESSMENT/RE-ASSESSMENT & DIAGNOSIS*
  - Percent of patients receiving physical activity assessment (physical activity history: type, duration, frequency)
  - Food/nutrition related data (serving sizes, calorie intake, carbohydrate intake, fiber intake, protein intake, food or beverage intake, low nutrient density food intake, meal or snack pattern, frequency of food eaten away from home, food prep methods)
  - Percent of patients with assessment of nutrition knowledge, attitudes, or beliefs
  - Percent of patients with documented obstetric history, including previous GDM
  - Percent of patients with documented risk factors or family history of diabetes
  - Percent of patients with nutrition diagnosis resolution
  - Percent of patients with improving nutrition diagnosis
- *NUTRITION INTERVENTION*
  - Percent of patients receiving physical activity guidance (type, duration, frequency, safety precautions with pregnancy)
  - Percent of patients receiving guidance on gestational weight gain recommendations
  - Percent of patients receiving at least 3 MNT encounters for GDM while pregnant
  - Percent of patients scheduled/attended at least one postpartum MNT visit related to GDM
  - Percent of patients with nutrition prescription documenting energy and macronutrient recommendations
  - Percent of patients receiving nutrition prescription that specifies protein goal (e.g., 1.1 g/kg BW)
  - Percent of patients receiving nutrition prescription that specifies meal and snack distribution
  - Percent of patients receiving counseling on breastfeeding promotion
  - Percent of patients receiving counseling on lifestyle behavior and prevention of type 2
  - Percent of patients where collaboration/coordination of nutrition care is an intervention strategy
- *NUTRITION MONITORING AND EVALUATION*
  - Percent of patients with changes in knowledge, or motivation, or readiness to change documented by stage of change
  - Percent of patients with understanding of treatment plan
  - Gestational weight gain (e.g., % patients with gestational weight gain in alignment with IOM recommendations)
  - Percent of patients requiring pharmacologic therapy for GDM management
  - Percent of patients with GDM and a diagnosis of hypertensive disorder in pregnancy
  - Biochemical data
  - Glucose records (% SMBG, fasting in range; % SMBG, postprandial in range)
  - Ketone records (if indicated)
  - Percent of patients with documented delivery type, infant birth weight, or adverse complications
  - Percent of patients requiring c-section delivery
  - Percent of patients with infant birth weight > 9 lbs. at birth
  - Percent of patients who receive postpartum glucose testing

GUIDELINE IMPLEMENTATION CHECKLIST

Sites are encouraged to use this checklist to track activities related to guideline implementation.

Step	Notes	Completed (no/yes)	Date of Completion
RDN and team training or knowledge acquisition			
Form an implementation/QI team, including external stakeholders/patients when relevant			
Draft an aim statement – what do you want to accomplish?			
Assess current practices at baseline and identify gaps between current and recommended practice			
Assess barriers of guideline implementation or use (e.g., patient, professional, organizational, system, etc.)			
Describe the problem, prioritize problem(s) related to guideline implementation			
Identify strategies that will be effective and best suited for guideline implementation			
Identify and assemble resources needed for implementation			
Develop an implementation plan (e.g., roles, responsibilities, strategies, milestones, time frames, and measures to evaluate implementation)			
Notify dietitians and other clinical providers of guideline (emails, letter, newsletter, discuss at meetings)			
Guideline implementation tools (e.g., patient teaching aids, caregiver resources, point of care tools (checklists, decision aids, algorithms))			
Implement your action plan (the do-phase)			
Collect data as you implement your action plan and study your efforts (e.g., monthly QI progress worksheets to document problems, unexpected effects, trends, or general observations)			
Reflect on plan and outcomes, re-examine processes and if applicable plan, adopt, adapt, or re-start the planning process			

Adapted from the Guideline Implementation Planning Checklist[12]

Note: Plan, Do, Study, Act (PDSA) cycles allow for testing a change and determining what modifications may be needed in an *ongoing* process.

[12] Gagliardi AR, Marshall C, Huckson S, James R, Moore V. Developing a checklist for guideline implementation planning: review and synthesis of guideline development and implementation advice. *Implement Sci*. 2015;10:19. doi:10.1186/s13012-015-0205-5

PDSA WORKSHEET INSTRUCTIONS

Sites will submit a PDSA worksheet ***before and after*** each test cycle.

Sites can access a PDSA worksheet template by visiting the IHI Website.[13]

General Guidance:

1. Sites will use the PDSA worksheet to PLAN a PDSA cycle.
    a. Revisit your approved aim and document your planned change to test along with your prediction of what may happen.
    b. Sites should fill in the "PLAN" sections of the worksheet to describe the plan for change and plan for data collection.
    c. Submit the completed worksheet for study approval before starting a test cycle.
    d. When submitting your worksheet, please include the site facility number.
2. Sites will also use the PDSA worksheet to record findings from the DO-STUDY-ACT phases.
    a. Sites will fill in the "DO-STUDY-ACT" sections of the worksheet to document what happened and PDSA outcomes.
    b. As a reminder, do not include any protected health information in your summaries.
    c. Sites will submit the completed worksheet after finishing a test cycle.
    d. When submitting your worksheet, please include the site facility number and the start and end date of the test cycle.

[13] Institute for Healthcare Improvement. *Plan-Do-Study-Act (PDSA) Worksheet*. IHI website. https://www.ihi.org/library/tools/plan-do-study-act-pdsa-worksheet
