# Supplementary material for: Implementation of clinical practice guidelines using the Plan–Do–Study–Act framework: The methodology and experiences of the Academy of Nutrition and Dietetics Health Informatics Infrastructure Registry Study on gestational diabetes mellitus
Source: Nutr Clin Pract. 2025 Oct 2;40(6):1465–82. doi: 10.1002/ncp.70043 (PMC12590319; doi:10.1002/ncp.70043)
Supplement: Supplementary file 2 — Supplemental File 2. [file NCP-40-1465-s001.pdf]

QUALITATIVE CODEBOOK

GDM REGISTRY STUDY

Navigating Context, Capacity, and Possibility

Description: How individuals make sense of their roles, systems, and constraints while still identifying space for possibility within limited bandwidth and complex settings.

Sample Reflection: RDNs work independently but often face professional silos, affecting clarity of roles and collaboration. Stepping back to reflect is important - enables providers to question routine practice and identify areas for change.

Codes:
- Implementation User Characteristics
- Professional Isolation & Role Clarity
- Status Quo [reflection]
- Collaboration & Communication
- Limited Bandwidth [time constraints, competing priorities]
- Inner Setting vs. Outer Setting

Example Quotes:
Professional Isolation & Role Clarity
- I would say primarily it was RD focused, me focused, and I'm the only one.

Status Quo
- As far as dietitians, we're really good at being able to do our job without a whole lot of help, and sometimes we just get into our rut of things
- I know that it's important not to just rest on your laurels or go with a status quo of what you always have been doing.

Limited Bandwidth
- Definitely our tight schedule and the lack of time for communication, meaning our whole day here is patient focused, so we don't get a lot of time to discuss with our clinical staff or office staff. Our meetings have been cut and are going to be less frequent…the agendas are packed with pressing information.
- One of our issues is staffing, and my team, as of dietitians, we're pretty booked. There's a quite lengthy wait list to have folks come in and see us.
- Implementing changes to documentation policies can be, I would say, somewhat bureaucratic in the sense that it takes a while, so I think that would probably be the other biggest barrier, is just simply put time.

Outer Setting
- I know that we are supposed to be doing more QI just as a department. And with COVID and everything, it became sort of a back burner thing.
- Some of those bigger things would be outside of myself, and so I have no control over it. And nothing might change because of it.
- It's kind of like you bring in insurance, you bring in just people's schedules, you bring in all these different factors on what's covered and what is done, and you quickly realize, 'Okay, nope. We're moving on to different things.'

Sizing Up Change: Fresh Eyes on Familiar Practices

Description: Involves reflecting on existing practices, applying standards/guidelines, and brainstorming what change might look like in a local context.

Sample Reflection: Participants reflected on aims while considering real world limitations of implementation. They explored guideline implementation as a group to determine what was possible and feasible.

Codes:
- Honest Reflection & Fit [Fit = Feasibility, Acceptability]
- Using Guidelines or Standards to Differentiate Practices
- Brainstorming

Example Quotes:
Honest Reflection & Fit

- Humbling…what can we actually do, what is realistic, what do we have control over, who else is involved, or that patients will respond to.
- Couple different guidelines that we were kind of entertaining, not so much that it couldn't be done in a longer span of time.
- It [referring to a guideline recommendation for 3 MNT visits] was going to involve a lot more people with changing schedule ... Getting schedulers on board, insurance, just even factoring in our schedules when we were only there twice a week. Do we have room on those days to see people for a third visit? Versus the ones we ended up doing were tangible things that XXX and I could do and not have to rely so much on other people to help make sure they got done.

Using Guidelines or Standards to Differentiate Practices

- That was nice to just to see, to actually kind of weed out, okay, we are doing this well versus this is an area we could grow, that was pretty eye opening.
- Having the GDM guidelines listed out was so helpful, and was really fun to see what we were already doing and then the areas where we maybe weren't quite meeting those recommendations and trying to see, how could we try to get us closer to that.

Brainstorming

- What started out as a huge list quickly and dwindled the like, okay, I think we can reasonably work this kind of thing too. Yeah, there's always so many other factors involved.

Plan Phase: Translating Shared Vision to Action

Description: Describes the alignment of team understanding, goal setting, and infrastructure to move from intention to coordinated, practical action. Aim development and PDSA worksheets were valued by participants.

Sample Reflection: Aligning on aims was important for collective commitment and collaborative action. Tools, such as PDSA and facilitation via working with the principal investigator supported planning.

Codes:
- Sense Making
- Setting Aims-Developing Aims
- Identifying Outcomes
- Champion
- Buy-in & Acting Together [teamwork, communication, collaboration]
- Planning Infrastructure [Structure, Facilitation, Planning Tools such as aim development, PDSA worksheets, QI manual]
- Operationalizing the Plan [Processes-Redesign]

Example Quotes:

Sense Making

- After we both did those training modules and went into the QI phase, we sat down and kind of talked through what stood out to us, taking some notes, and we kind of talked through what you know a tangible change that we could make. We actually both really easily agreed on protein being what we could what we could bring in.
- Just putting it out on paper like, this is our goal. This is how much we're actually meeting it, this is how much we'd like to meet it, and where that gap is.

Setting Aims

- I do think it's helpful kind of knowing where we're going and what our goal is.
- I tried to set new aims, I would think, okay, within the registry database, the terminology, what could I be using as an outcome.
- Can I look at outcome data and see that that's making a difference?

Planning Infrastructure

- The QI workbook was very helpful, because I felt like because that was all new to me… I would say just having almost step by step and a lot of ideas was very helpful…
- I felt like the workbook was helpful as a resource.
- I think doing the actual PDSA worksheets, coming up with aims, putting it on paper, developing a plan of action to what we already knew was a gap in our care.
- [referring to PDSA] get down to details and the actual what’s next steps.
- I appreciate all your time and effort and communication and hard work for keeping us on track and keeping encouraging us. Because we definitely have time as a big barrier in our schedules. And you kept your patience and everything with dealing with us not quite getting it or getting up to speed. We really appreciate it. [reflects facilitation, facilitator role]

Do Phase: Boots on the Ground and Eyes to the Sky

Description: Captures the real-time work of implementation—testing, adapting, and staying centered on patient care while navigating workflow and challenges.

Sample Reflection: Teams used PDSA to keep work moving forward and refine improvements overtime. Optimizing workflow, particularly communication can improve efficiency. Dietitians prioritized patient centered care while balancing aims.

Codes:
- Iterating Practice Changes [Implementation, De-implementation, Testing, EBP is Ongoing]
- Staying Patient Centered
- Adherence to Guidelines [checking work, comparing practice to guidelines, mindfulness]
- Dealing with Challenges & Problem Solving
- Workflow Efficiency

Example Quotes:

Iterating Practice Changes

- Kind of keeping it simple, you know, looking at what you really need to improve on, and you know, knowing the guidelines.
- I think it’s good because you can kind of adjust and build on what you’re already doing. So, I know I could have switched to a different topic, but I think it was good to kind of focus on breastfeeding, but kind of build off of that and seeing what works or how to make it better I think was helpful.

Staying Patient Centered

- Our mission is always patient first, we're always trying to put the patient first.
- Just where the patient is, you know, can prepare and think you're going to have one conversation and then it turns completely into something else.
- So, zoning in on what is very important and which, because you can only focus on so much. I mean these moms are, they have full-time jobs, they have other kids in the household and now they're gestational, they have gestational diabetes and now they have to do above and beyond what they have done in the past in their previous pregnancies.
- I think it’s important to give the patient the information so they can make their own decision.

Adherence to Guidelines

- I found myself really double checking the research on things, really reading the data a lot more.
- Helpful that you had all of the applicable standards of care, whether it be from ADA, ACOG guidelines, or any of the applicable governing bodies that deal with this specialty. That was helpful to have that all in one place. I have that in a binder, and so I use those resources,
- I have been able to really just double check my work and really make sure that I am providing the care according to standards and practices.

Workflow Efficiency

- But that whole kind of wanting to get everybody's input and how would it be best formatted for what we need it for, that definitely took a little bit more time than I think I thought I would.
- Kind of waiting for people or communication… I think it just kind of slowed everything down.

Study Phase: Learning from Data and Experiences

Description: Encompasses feedback loops and reflection on practice using data, structured communication, and shared learning across roles.

Sample Reflection: Data and study prompts, such as PDSA reporting kept teams accountable. Shared documentation tools improved consistency in care.

Codes:
- Data Driven Accountability [Using data to learn & improve]
- Speaking the Same Language [using structured language to document care, consistent messaging among providers]
- Experiences & Effectiveness

Example Quotes:
Data Driven Accountability

- When you're working on something like a study. Well, it's not like we want to do good, of course we do, but we want to find out where our weaknesses are, where we want to improve. It's not like you're doing a study so you can come out on top or the best, no. It's to make sure that we know we can improve and we're lacking. it really opened up everybody's mindset.
- I think this study really elevated that, let's say criteria for success.
- We're kind of scheduling an appointment with ourselves to check out the guidelines. How are we doing? Can we reassess? I feel like the study provided a lot of structure, which in just daily practice it's easy to let go of that if you don't have those prompts.
- Help stay on track or keep everyone who's involved with it on the same page…really helpful just to keep things moving forward.

Speaking the Same Language

- Being able to keep that terminology cohesive between all of us because it can be confusing when you're following up on somebody else's patient and it's a different terminology… I think that's been super helpful for all of us.
- I think it was definitely good to keep the data standardized so everyone is using the same terminology.
- It's definitely helpful to make sure that we're following up on the right things and tracking the same measures.
- We adjusted our template for our note because it didn't include the breastfeeding portion, so we included that in there now so everyone can use that.

Experiences & Effectiveness

- This [study] has helped us to discuss it and talk about it. I think it's improved our conversation about our program.
- I felt like I'm a stronger clinician and an educator because of it.
- People seem to actually usually increase their protein a good bit more than what they came in.
- During class, you know, they [patients] seem to be interested.

Act Phase and Beyond: Seeing the Ripple, Strengthening the System

Description: Highlights how teams embed changes, share lessons, and look ahead—strengthening systems through integration and iterative growth.

Sample Reflection: Dietitians expressed an interest in continued improvement and intentions to continue using tools, such as PDSA cycles in the future. Integration helped with consistency among the team but also fostered spread to other members of the healthcare (e.g., other dietitians, nurses, diabetes educators). Dietitians were proud of what they did, but were eager to continue to improve.

Codes:
- Building Relationships for System Learning [sharing lessons learned]
- Integration fosters sustainability and spread + strategies that worked [tools they would use again]
- Perceived benefits - professional value
- Iterative improvement mindset - future plans

Example Quotes:

Building Relationships for System Learning

- I think for me, I would really like to just see a little bit more cohesion or communication within our department as it pertains to our nurse team and the dietitians.
- We would really love to do some more outreach, talking to the OBs more and getting the word out there.
- Sharing what we've done with in this study already with the rest of the team, there is a large number of outpatient dietitians and anyone can see a GDM diagnosis. I definitely think passing along the information and the resources would be the first thing.
- I think system-wide too, we need to show everyone this resource and try to make sure we're consistent with using that.

Integration fosters sustainability and spread + strategies that worked

- I think it definitely helped to add it into our note template, because that's kind of the outline I have in my head when I'm going through with my consults…
- We added it in to the template for our note…so we're all kind of talking about the same thing.
- We added it to one of our smart phrases. It's actually a nice reminder.
- The workbook where it had the guidelines in there and then it also had a few different spots where it kind of made you think about your program…would be a realistic first step.
- EAL, the library, that was really helpful, the guidelines.
- I think for me, the PDSA cycles and the worksheet, like I said earlier, how it helps you really break it down into what specifically is next and who is going to be responsible.

Iterative Improvement Mindset

- I think the study has really helped us realize that this is a focus that we can just keep working on until we see that it's working.
- See, okay, what's next? What is the next area we can work on to get us closer to fulfilling those guidelines completely?

Notes:

A list of semantic codes was derived from the transcripts by the principal investigator. Initially 52 codes were generated and clustered into 6 themes developed from the analysis. During the 2nd round of coding, codes were organized leading to 32 codes; and in the 3rd round of coding, codes were further consolidated for a total of 28 codes under 6 themes. ChatGPT was used as a language-based tool to enhance clarity of the final theme names, codes, and descriptions. No content was generated independently of researcher oversight. AILYZE, an artificial intelligence–powered qualitative analysis tool was used to generate two independent thematic analyses (1) based on AI generated coding and (2) based on the researcher developed codebook. The AI generated thematic analysis described 3 themes and 12 subthemes. When our codebook was applied, the AI tool coded excerpts similar to the coding completed by the researcher. AILYZE augmented our analysis to assess alignment of coding and enhance analytic reflexivity. The researcher derived codebook and themes were used for the final results.

OpenAI. OpenAI. ChatGPT [large language model]. ChatGPT-4. Updated 2023.

AILYZE I. AILYZE. www.ailyze.com
